# The eIF2 kinase GCN2 directs keratinocyte collective cell migration during wound healing *via* coordination of reactive oxygen species and amino acids

**DOI:** 10.1016/j.jbc.2021.101257

**Published:** 2021-09-29

**Authors:** Rebecca R. Miles, Parth H. Amin, Miguel Barriera Diaz, Jagannath Misra, Erica Aukerman, Amitava Das, Nandini Ghosh, Tanner Guith, Michael D. Knierman, Sashwati Roy, Dan F Spandau, Ronald C. Wek

**Affiliations:** 1 Department of Biochemistry & Molecular Biology, Indiana University School of Medicine, Indianapolis, Indiana, USA; 2 Department of Dermatology, Indiana University School of Medicine, Indianapolis, Indiana, USA; 3 Department of Surgery, Indiana University School of Medicine, Indianapolis, Indiana, USA; 4 Indiana Center for Regenerative Medicine and Engineering, Indiana University School of Medicine, Indianapolis, Indiana, USA; 5 Laboratory for Experimental Medicine, Eli Lilly and Company, Indianapolis, Indiana, USA; 6 Richard L. Roudebush Veterans Administration Medical Center, Indianapolis, Indiana, USA

**Keywords:** Gcn2 eIF2 kinase, translation control, keratinocyte collective cell migration, wound healing, CST, Cell Signaling Technology, DE, differentially expressed, ECM, extracellular matrix, eIF2, eukaryotic initiation factor 2, HDW, high-density wounding, IPA, Ingenuity Pathway Analysis, ISR, integrated stress response, KCCM, keratinocyte collective cell migration, KO, knockout, NHK, normal human epidermal keratinocyte, ROS, reactive oxygen species

## Abstract

Healing of cutaneous wounds requires the collective migration of epithelial keratinocytes to seal the wound bed from the environment. However, the signaling events that coordinate this collective migration are unclear. In this report, we address the role of phosphorylation of eukaryotic initiation factor 2 (eIF2) and attendant gene expression during wound healing. Wounding of human keratinocyte monolayers *in vitro* led to the rapid activation of the eIF2 kinase GCN2. We determined that deletion or pharmacological inhibition of GCN2 significantly delayed collective cell migration and wound closure. Global transcriptomic, biochemical, and cellular analyses indicated that GCN2 is necessary for maintenance of intracellular free amino acids, particularly cysteine, as well as coordination of RAC1-GTP-driven reactive oxygen species (ROS) generation, lamellipodia formation, and focal adhesion dynamics following keratinocyte wounding. *In vivo* experiments using mice deficient for GCN2 validated the role of the eIF2 kinase during wound healing in intact skin. These results indicate that GCN2 is critical for appropriate induction of collective cell migration and plays a critical role in coordinating the re-epithelialization of cutaneous wounds.

Although healthy individuals heal cutaneous wounds without complication, there are many patients whose wounds do not resolve and require long-term care. In fact, chronic cutaneous wounds are an epidemic that are frequently associated with diabetes, aging, and poor nutrition (1, 2, 3). Wound healing occurs in four sequential phases: hemostasis, inflammation, proliferation, and maturation. An important process during the proliferation phase is re-epithelialization that involves keratinocyte collective cell migration (KCCM) where sheets of connected cells migrate in concert to cover the wound bed (4). If these phases are dysregulated or do not occur in an orderly fashion, a wound will not properly heal. A chronic wound is defined as one that does not heal within 3 months. Chronic wounds are most often arrested in the inflammation phase, stalling keratinocyte migration and the subsequent sealing of the wound bed (5). Because re-epithelialization is impaired in many types of chronic wounds, further research is warranted to find novel therapeutic strategies to restore and enhance epithelial barrier formation (6).

To study keratinocyte epithelial migration during wound healing, wounds are often modeled by “scratching” or removing sections of confluent sheets of differentiated keratinocytes and monitoring KCCM in two-dimensional cell culture (7, 8, 9). For KCCM to occur and close a wound, cells must have physical connections that remain intact during migration and direct the restructuring of the cytoskeleton to provide a direction to their unified movement. Furthermore, KCCM requires proper preparation of the extracellular matrix (ECM) to create a substrate upon which the collective sheet can effectively migrate (8). These distinct and complex tasks have been assigned to two types of cells in the KCCM: leader and follower cells (10, 11). It is suggested that generation of reactive oxygen species (ROS) in keratinocytes at the leading edge of the wound is critical for directional cell migration through remodeling of the cytoskeleton to form lamellipodia and filopodia (12, 13).

Disruption of normal keratinocyte–keratinocyte interactions during wound formation causes a major mechanical stress within the epidermis. An important mechanism for cell remediation of environmental stresses involves phosphorylation of the α subunit of eukaryotic initiation factor 2 (eIF2α-P). Multiple eIF2α kinases respond to distinct stresses, including amino acid depletion and UVB irradiation (GCN2, EIF2AK4) and accumulation of unfolded proteins in the endoplasmic reticulum (PERK, EIF2AK3) (14, 15). eIF2α-P reduces delivery of initiator tRNAs to ribosomes, sharply decreasing bulk protein synthesis, which lowers nutrient consumption and facilitates reprogramming of gene expression to alleviate stress damage. Accompanying the global reduction in protein synthesis, eIF2α-P also directs preferential translation of key genes that are critical for stress remediation, such as the transcription factor ATF4, which directs adaptive reprogramming of gene expression. Therefore, eIF2α-P induces both translational and transcriptional modes of gene expression to mitigate stress damage. Because eIF2α-P responds to different stressors to induce translational control, the pathway is referred to as the integrated stress response (ISR) (16). It is important to emphasize that while ATF4 is central for implementation of gene expression in the ISR in response to many stresses, there are certain stresses, such as UVB irradiation in keratinocytes (17, 18), where the adaptive functions of the ISR occur independent of ATF4 through other target genes. Therefore, GCN2 in the ISR is suggested to interface with multiple effector target genes to tailor adaptation to different stresses. Along with activation of eIF2α kinases, the magnitude and duration of eIF2α-P and the accompanying translation control are regulated by type 1 protein phosphatase *via* two targeting subunits, CReP (PPP1R15B) or GADD34 (PPP1R15A) (15, 19). While CReP is suggested to be constitutively expressed, GADD34 is itself induced by the ISR, creating a negative feedback loop that serves to lower eIF2α-P and restore protein synthesis upon resolution of stress damage.

Given the important roles for GCN2 in skin homeostasis and the roles of eIF2α-P in managing environmental stress, we addressed the role that GCN2 and the ISR play in KCCM during re-epithelization of cutaneous wounds. Using a combination of biochemical, genetic, and cellular approaches in cell culture and mouse model systems, we show that GCN2 and its attendant gene expression are important for appropriate management of amino acids, ROS generation, lamellipodia formation, and focal adhesion dynamics that are central for KCCM and optimal wound healing.

## Results

### Role for GCN2 in KCCM and wound healing

**Figure 1:**
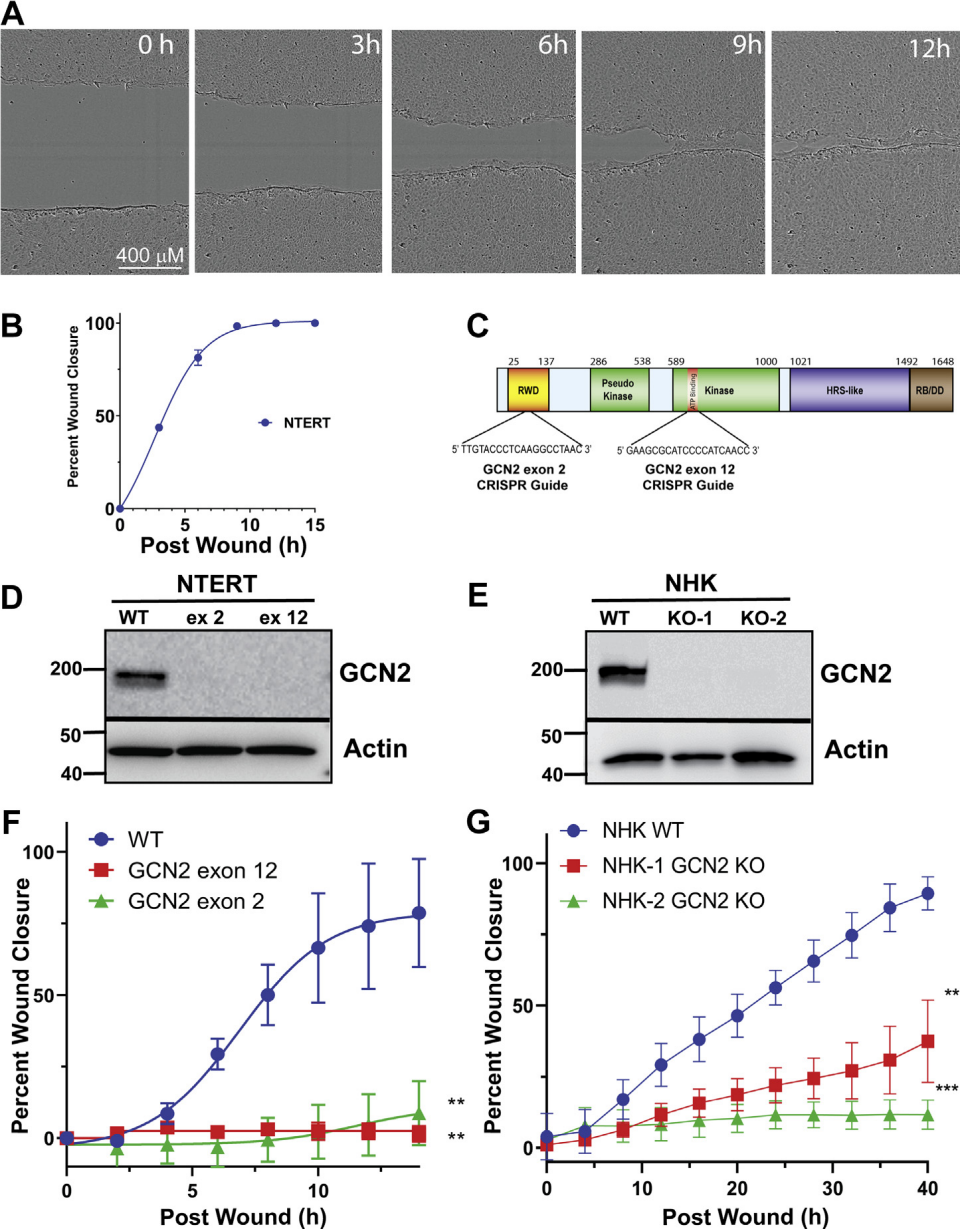
**GCN2 facilitates collective cell migration in cultured human keratinocytes during wounding.***A*, NTERT human keratinocytes were subjected to wounding and wound closure was monitored by an IncuCyte ZOOM Live-Cell Analysis System. Images show closure of the wound for up to 12 h post wounding. *B*, percent wound closure of NTERT cells over time. *C*, CRISPR/CAS9 guide design for targeting *GCN2* exon 2, which corresponds to the N-terminal RWD region, or exon 12 that encodes the ATP binding site in the kinase domain of GCN2. Efficiency of CRISPR/CAS9 GCN2KO was assessed by immunoblot measurements of GCN2 protein in (*D*) pooled NTERT cells edited with exon 2 or exon 12 sgRNA and (*E*) two independent pools of normal human keratinocytes (NHK) cells edited with the RNA guide targeting exon 12. Wild-type (WT) NTERT and NHK indicates cells with control sgRNA. Representative of three independent immunoblots. *F* and *G*, wound closure over time in control sgRNA WT and GCN2KO NTERT (n = 4) and normal human keratinocytes NHEK cells (n = 6). WT *versus* KO One-way ANOVA Tukey's multiple comparisons test. Error bars are SD and ∗∗ represents *p* < 0.01 and ∗∗∗*p* < 0.001.

The use of NTERT human keratinocytes is a well-established model to study skin biology and KCCM (17, 18, 20, 21, 22). KCCM during wound healing was measured using an IncuCyte Zoom instrument. Upon uniform scratch wounding of the NTERT cells with a WoundMaker tool, sequential time-lapse images were taken at regular intervals to show that the differentiated keratinocytes move forward into the wounded space as a sheet of cells (Fig. 1, *A* and *B*). These results confirm that our model recapitulates KCCM (23). Given that complete closure of the wound can occur within 9 h, this result indicates that KCCM was a primary result of migration and not proliferation since NTERT keratinocytes have a doubling time of 24 to 30 h *in vitro* (24).

We addressed the role of GCN2 in KCCM by using CRISPR/CAS9 and guide RNAs targeting exon 2, which encodes the N-terminal RWD domain of GCN2, or exon 12, a region encoding the critical lysine reside in the kinase domain (Fig. 1*C*). The guide RNAs edited the *GCN2* (*EIF2AK4*) loci, creating a pool of knockout (KO) keratinocytes effectively lacking GCN2 protein expression (GCN2KO) (Fig. 1*D*). Early passage GCN2KO cells were used in all experiments. Using wound healing assays, we observed a marked reduction in wound closure of the GCN2KO cells compared with the WT NTERT controls (Fig. 1*F*). We confirmed these results using two different primary normal human epidermal keratinocytes (NHK) that were isolated from foreskin tissue from separate donors (25). The sgRNA targeting exon 12 was used to delete *GCN2* in NHKs derived from two separate individuals (Fig. 1*E*), and analysis of wound closure in both donor GCN2KO cells showed delayed closure (Fig. 1*G*). We conclude that GCN2 facilitates KCCM in during wounding.

### Wounding activates GCN2 and the eIF2*α* kinase is required to sustain the ISR

**Figure 2:**
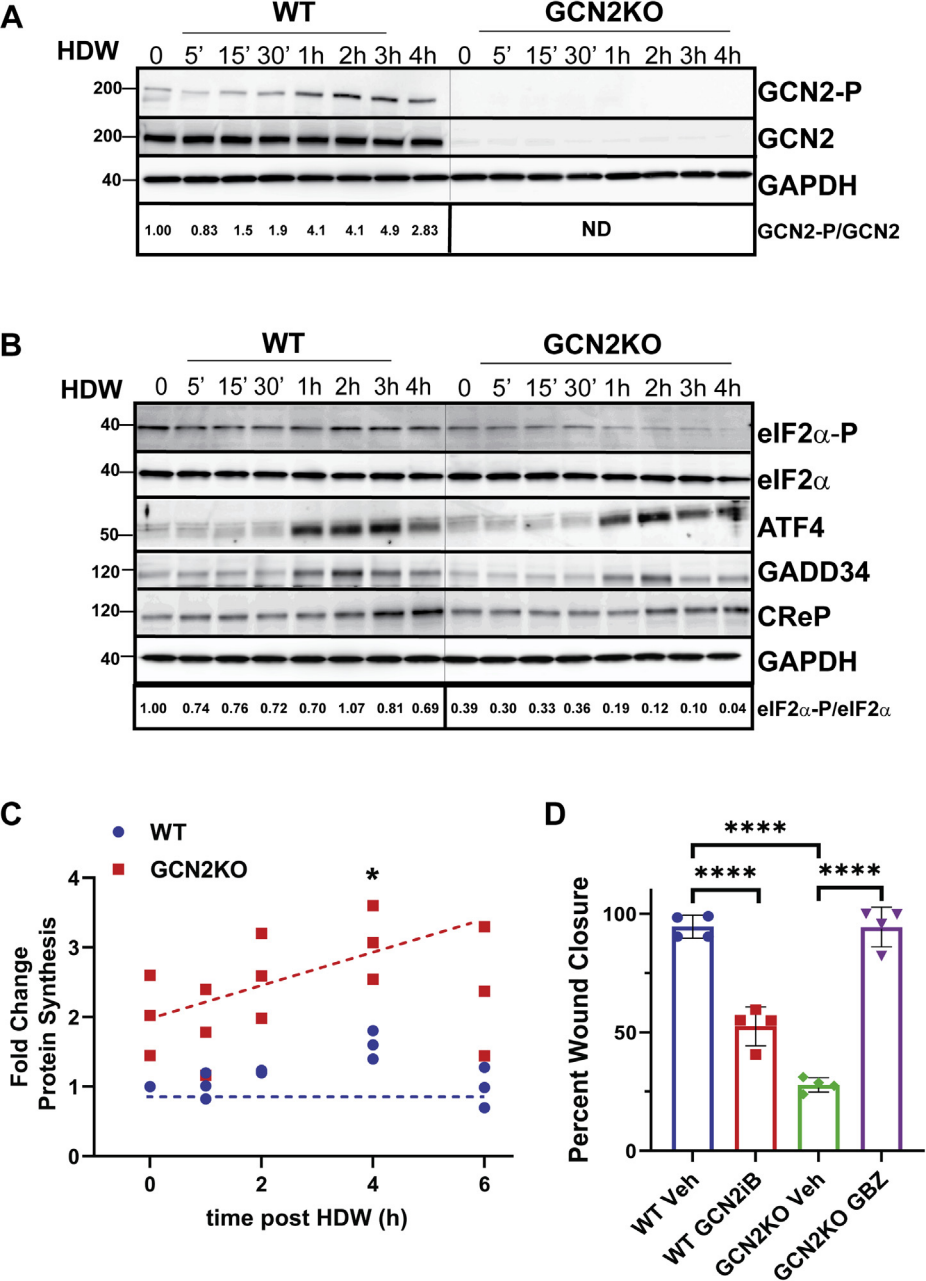
**High-density wounding induces GCN2-P and sustains eIF2α-P during keratinocyte collective cell migration.***A* and *B*, WT and GCN2KO cells were subjected to HDW and lysates were collected at specified times after wounding for immunoblot analyses. Immunoblot analyses using antibodies specific to the indicated proteins. Immunoblot panels for WT and GCNKO cells for each protein were carried out in one experiment to allow for direct comparisons. *A*, fold change for phospho-GCN2 is indicated at the *bottom* of the immunoblot panels. “ND” indicates that total and phosphorylated GCN2 was no detectable in the GCN2KO in the immunoblot analyses. *B*, fold change for phospho-eIF2α is indicated at the *bottom* of the figure panels. *C*, WT and GCN2KO NTERT cells were collected after up to 6 h following HDW. Zero hour indicated no wounding. Thirty minutes prior to harvest, 1 μM puromycin was added to the cultures to label nascent polypeptides. Lysates were prepared, subjected to SDS-PAGE, and puromycin-labeled nascent polypeptides were measured by immunoblot analysis using antibodies specific to puromycin. A representative blot is included as Fig. S2. Levels of bulk protein synthesis as reflected in puromycin tagged proteins in the immunoblots are shown as fold change relative to WT cells not subjected to HDW. One-way ANOVA Dunnet’s multiple comparisons, (n = 3) ∗*p* < 0.05. *D*, WT NTERT cells were treated with 5 μM GCN2iB at the time of wounding and percent wound closure was measured at 16 h. GCK2KO cells treated with 1 μM guanabenz at the time of wounding or vehicle and percent wound closure was measured after 16 h. One-way ANOVA Tukey's multiple comparisons test (n = 4); Error bars are SD and ∗∗∗∗*p* < 0.0001.

To determine the molecular consequences of depleting GCN2 in the initiation of KCCM following wounding, we developed a technique called high-density wounding (HDW). This method enhances the numbers of keratinocytes in proximity to the scratch wound using tips attached to a multichannel pipettor to make parallel scratch wounds on tissue culture plates (Fig. S1). Using constant pressure, the tips were drawn across the well once in both horizontal and then vertical directions. The HDW method created a regularly spaced grid of wounds spaced 5 mm apart, enriching the number of leader cells to organize the initiation of KCCM. Given that measurable progression of the leading front can be detected between 2 and 4 h post wounding, we collected cell lysates at intervals from 5 min to 4 h post wounding to capture the initiation, amplitude, and duration of the ISR. Control WT and GCN2KO NTERT cells were differentiated into epithelial sheets for 48 h before being subjected to HDW. Immunoblot analyses of WT NTERT lysates revealed that wounding leads to activation of GCN2, as measured by GCN2 phosphorylation, within 30 min to 1 h post injury (Fig. 2*A*). Of importance, the WT NTERT cells showed enhanced eIF2α-P in the unwounded cells compared with GCN2KO, and these levels were sustained in the WT cells throughout the wounding time course (Fig. 2*B*). By comparison, eIF2α-P was reduced in the unwounded GCN2KO cells and was sharply depleted after 1 h of wounding. These results indicate that activated GCN2 during wounding is necessary to sustain eIF2α-P levels and is critical for KCCM.

ATF4 has a prominent role in the implementation of the ISR during many environmental insults. ATF4 expression was increased following wounding; however, elevated ATF4 protein levels occurred independent of the functional status of GCN2 (Fig. 2*B*). While eIF2α-P is typically important for translation induction of *ATF4*, there are other reported mechanisms involving changes in *ATF4* mRNA synthesis and turnover and ATF4 protein stability, which significantly contribute to enhanced ATF4 protein levels, and some these processes involve ancillary pathways (26, 27, 28, 29, 30, 31, 32). The role of ATF4 in the wound closure will be furthered addressed below. ISR-directed translation is controlled in part by negative feedback through induced expression of GADD34 and CReP. The increase in GADD34 and CReP expression following wounding was similar in both WT and GCN2KO keratinocytes. These results suggest that loss of GCN2 combined with increased GADD34 and CReP levels contributes to reduced eIF2α-P in wounded GCN2KO cells.

Bulk protein synthesis in WT and GCN2KO NTERT cells subjected to up to 6 h HDW was measured using puromycin labeling. There was about a twofold increase in protein synthesis in unwounded GCN2KO cells compared with control WT (Fig. 2*C* and representative blot in Fig. S2). Upon wounding, there was up to a threefold increase in global translation in GCN2KO keratinocytes after 4 to 6 h of HDW compared with WT cells. These results support the model that GCN2 and associated eIF2α-P contribute to lowered global protein synthesis following wounding.

Small-molecule inhibitors are frequently used as modulators of the ISR. For example, GCN2iB is a potent inhibitor of GCN2 (33), and guanabenz interferes with GADD34-directed dephosphorylation of eIF2α-P resulting in ISR activation (34). WT and GCN2KO NTERT cells were measured in the wound healing assay and treated with vehicle, GCN2iB, or guanabenz prior to and during the wounding assay. GCN2iB impaired wound closure of WT NTERT cells, whereas guanabenz compensated for the loss of GCN2 and restored KCCM in GCN2KO cells (Fig. 2*D*). Treatment of WT NTERT cells with guanabenz did not alter wound closure kinetics (data not shown). Similarly, treatment of GCN2KO cells with GCN2iB did not change KCCM following wounding (data not shown). These results further support the model that eIF2α-P by GCN2 is critical for wound closure.

### Loss of ATF4 does not disrupt wound healing

It is curious that wounding induced ATF4 expression in both WT and GCN2KO NTERT cells as previously we demonstrated that UVB-induced activation of GCN2 in NTERT keratinocytes does not lead to increased ATF4 expression (17, 18). We used CRISPR/CAS9 to delete an 800 bp region of ATF4 exon 4. As expected, ATF4 protein as measured by immunoblot was induced in WT NTERT cells upon exposure to thapsigargin, a potent ER stress agent, whereas there was minimal ATF4 in the knockout cells (Fig. S3*A*). Given this validation of the ATF4KO cell line, WT and ATF4KO NTERT cells were then tested in the wound healing assay. There was no difference in wound closure between WT and ATF4KO cells (Fig. S3*B*). However, treatment of the ATF4KO cells with GCN2iB delayed wound closure (Fig. S3*C*), showing that the knockout cells still required the GCN2-directed ISR for KCCM. These results are consistent with the results that induced ATF4 expression in NTERT cells is independent of GCN2 and suggests other target(s) for GCN2-directed wound closure in keratinocytes.

### GCN2 and transcriptome analyses in wound healing

**Figure 3:**
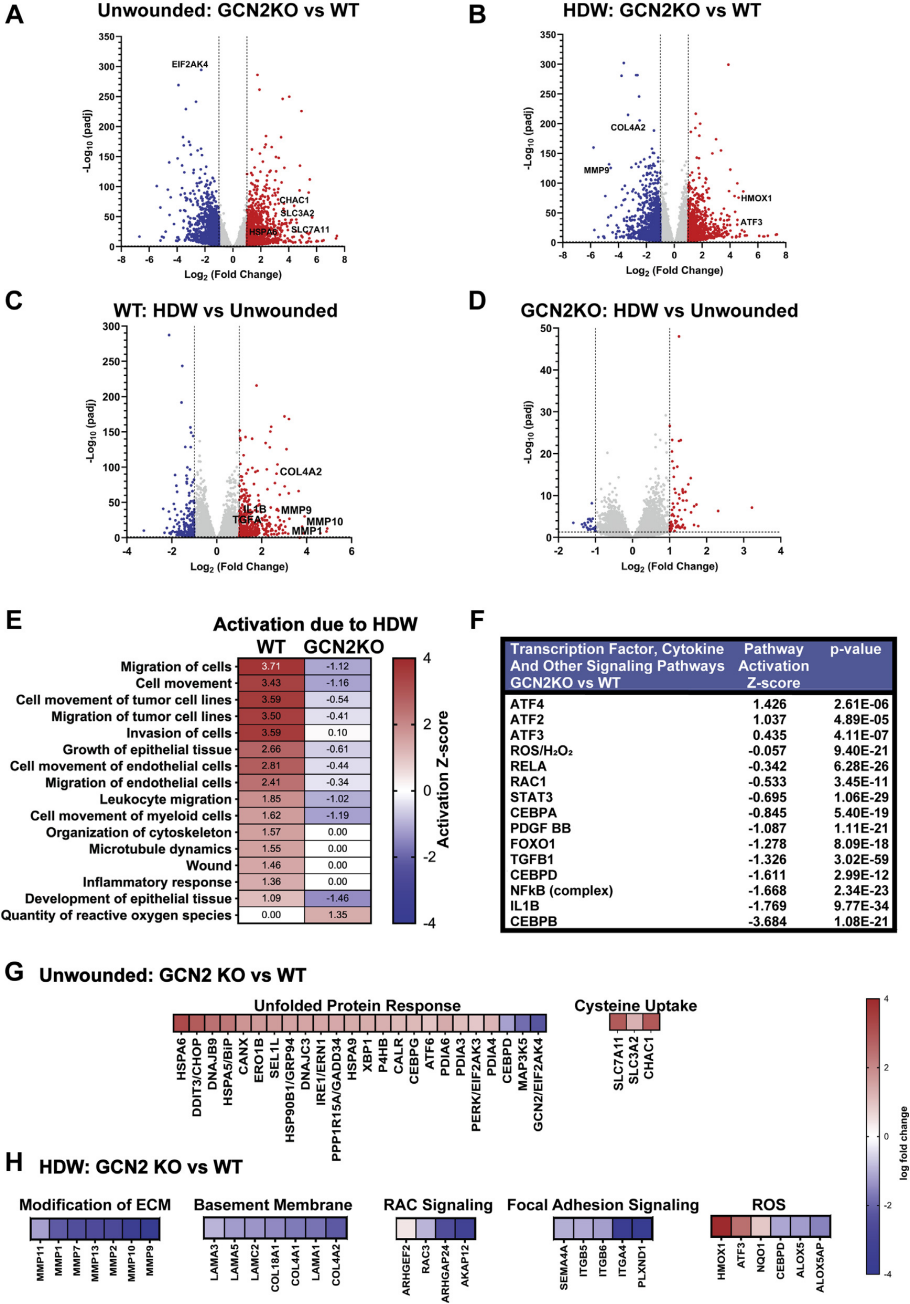
**Transcriptome profiling of wounded keratinocytes indicates altered expression of genes involved in cellular migration and the unfolded protein response.***A*–*D*, volcano plots illustrating log2fold change with p-adjusted value (−log base 10) between unwounded GCN2 KO *versus* WT (*A*), wounded GCN2 KO *versus* WT (*B*), WT wounded *versus* unwounded (*C*) and GCN2 KO wounded *versus* unwounded (*D*). *E*, heat map of activated molecular functions comparison between WT HDW-unwounded and GCN2 KO HDW-unwounded made using comparison tool of IPA. Heat map shows activation Z-scores with the scale showing the highest z scores in *red* and lowest in *blue*. *F*, table of upstream regulators of differentially expressed genes (DEG) in HDW GCN2 KO *versus* HDW WT group predicted using Upstream Regulator Effects Analysis tool of IPA. *G*, heat map of differentially expressed UPR genes and genes regulating cystine uptake identified in unwounded GCN2 KO NTERT cells. Data is depicted as log2fold change of genes in GCN2 KO unwounded *versus* WT unwounded group. The scale shows the highest log2fold change is denoted as *red* and the lowest is denoted as *blue*. *H*, heat maps illustrating a group of differentially expressed genes in wounded GCN2 KO NTERT cells. The genes are categorized based on distinct roles in different stages of cell migration. Data is represented as log2fold change of genes in GCN2 KO HDW *versus* WT HDW group. The scale to the *right of the panels* shows the highest log2fold change as denoted as *red* and the lowest represented as *blue*.

To delineate GCN2-dependent gene expression pathways in KCCM, we pursued an unbiased transcriptome analysis. WT and GCN2KO NTERT cells were subjected to HDW or left unwounded, and RNA was isolated and analyzed by RNA-seq (Table S1). DESeq2 was used to determine differentially expressed (DE) genes among different groups (35). Genes with log2fold change of ≥±1 and *p*-adjusted value of ≤0.05 were considered significant for further analyses. Expression of 1263 genes was significantly enhanced, and 1222 genes were significantly lowered upon deletion of GCN2 in NTERTs compared with unwounded WT NTERTs cells (Fig. 3*A*). With HDW, 1070 gene transcripts showed a significant increase, while 1209 were reduced in GCN2KO compared with WT (Fig. 3*B*). In WT NTERT cells, 400 gene transcripts showed a significant increase upon HDW, while 230 were lowered (Fig. 3*C*). By comparison, in GCN2KO subjected to HDW cells, only 73 gene transcripts showed a significant increase while 23 were significantly decreased, indicating that loss of GCN2 sharply diminishes the plasticity of mRNA expression upon wounding (Fig. 3*D*). These results show that wounding triggers significant changes in the transcriptome in WT keratinocytes and that loss of GCN2 significantly altered mRNA expression in both unwounded and wounded cells.

To distinguish which biological networks were affected by GCN2, we conducted pathway enrichment analysis using Ingenuity Pathway Analysis (IPA) software from Qiagen. HDW in the WT keratinocytes induced gene expression in wounding, cellular migration, cytoskeletal reorganization, and inflammation (Fig. 3*E*). By comparison, there were negative activation scores for these biological functions in wounded GCN2KO cells. Of importance, HDW in the GCN2KO cells enhanced genes involved in generation of ROS. Next, we used the IPA Upstream Regulator Analysis tool to predict which transcriptional regulators were disrupted with deletion of GCN2 (Fig. 3*F*). There were increased activation scores for genes regulated by the ATF transcription factor family (*ATF2*, *ATF3*, *ATF4*) and reduced activation scores for genes regulated by the CEBP transcription factor family (*CEBPA*, *CEBPB*, *CEBPD*). Genes associated with wound-associated cytokine signaling (*PDGFBB*, *TGFB1*, *IL1B*) and inflammatory pathways (*RELA*, NFKB complex, *IL6*, *STAT3*) also had negative activation Z-scores in the GCN2KO cells. It is also noteworthy that genes associated with *RAC1* and hydrogen peroxide formation had significant negative activation Z-scores in GCN2KO cells (Fig. 3*F*). These results suggest that GCN2 is critical for appropriate programming of multiple transcription networks affecting diverse cell functions, including stress responses, inflammation, and ROS generation.

We also addressed differences in gene expression in unwounded WT and GCN2KO keratinocytes that could alter the capacity for wound healing prior to any wound-induced stress. In the unwounded GCN2KO cells, there was significant increased expression of genes involved in the unfolded protein response (UPR) compared with unwounded WT cells (Fig. 3*G*). This result may reflect the demand placed upon the ER in GCN2KO cells to synthesize and correctly fold proteins in a cellular state with reduced eIF2α-P and translational control. Analysis of amino acid transporters in the unwounded state revealed upregulation of cystine cotransporter genes, *SLC7A11* (*xCT*) and *SLC3A2*, in the GCN2KO cells *versus* WT (Fig. 3*G*). *CHAC1*, an mRNA encoding an enzyme that converts cytosolic glutathione into free cysteine, was also sharply enhanced in the GCN2-deficient keratinocytes. Import of cystine and maintenance of free cellular cysteine is critical for glutathione production and ROS management (36).

We also assessed changes in HDW upon loss of GCN2 for gene transcripts involved in KCCM, including ECM, cytoskeletal dynamics, and ROS (Fig. 3*H*). In a comparison between HDW GCN2KO *versus* WT keratinocytes, there were significant reductions in the levels of gene transcripts encoding key basement membrane proteins, *COL4A1*, *COL18A1*, laminin alpha chains, as well as many matrix metalloproteinases (MMPs). Expressions of key integrins *ITGA4*, *ITGB5*, *ITGB6*, and interacting transmembrane genes *SEMA4A* and *PLXND1* were also sharply lowered with deletion of GCN2, as were expressions of genes involved in RAC1 signaling. RAC1 coordinates ROS production, which is required for actin cytoskeletal rearrangements necessary for formation of leading edge lamellipodia. Among genes involved in ROS management in the GCN2KO cells, expressions of NRF2 target genes, *HMOX1*, *ATF3*, and *NQO1*, were significantly increased, suggesting an adaptive response against active oxidative stress occurred. However, other genes known to contribute to ROS generation, such *ALOX5* and *CEBPD*, were downregulated in GCN2KO cells. These results underscore that GCN2 is critical for appropriate management of multiple critical transcriptome networks involved in KCCM. These networks are suggested to be directed by GCN2, ensuring the ability of these cells to implement key phases in wound healing.

### GCN2 and cysteine maintenance during wound healing

**Figure 4:**
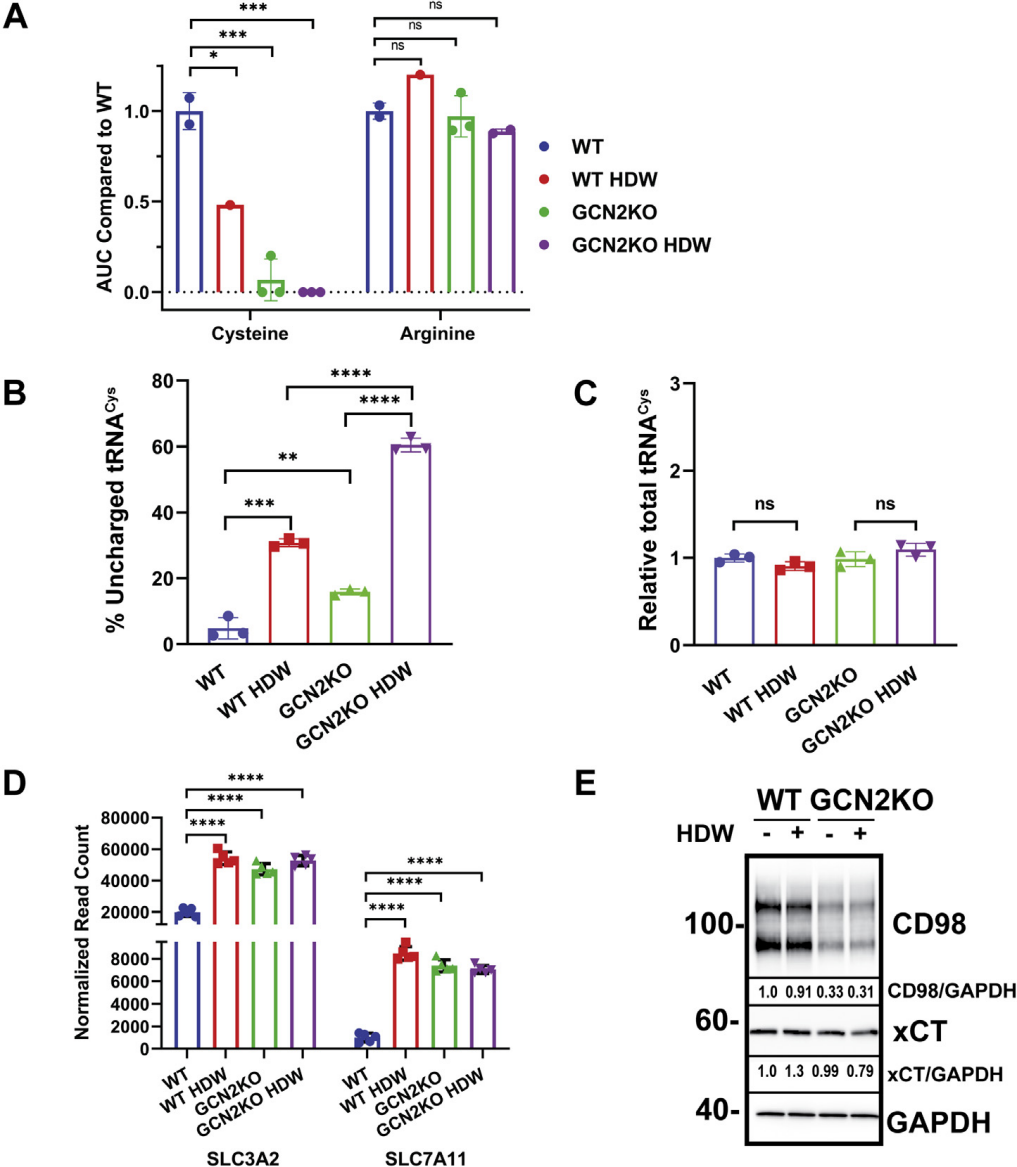
**GCN2 is required for maintenance of free cysteine levels in keratinocytes.***A*, quantitative CE-MS measurement of free cysteine and arginine levels in WT and GCN2KO cells harvested at 4 h postwounding. Area under the curve (AUC) values are shown relative to WT cells not subject to wounding. One-way ANOVA Tukey’s multiple comparisons. ∗*p* < 0.05, n.s. *p* > 0.05. *B*, levels of uncharged tRNA^Cys^ and (*C*) total tRNA^Cys^ were measured in WT and GCN2KO NTERT cells subjected to wounding. Two-tailed *t* test, ∗*p* < 0.05, ∗∗*p* < 0.01, ∗∗∗*p* < 0.001, ∗∗∗∗*p* < 0.0001 (n = 3). Error bars are SD. *D*, *SLC3A2* and *SLC7A11* gene transcripts were measured in unwounded WT and GCN2KO keratinocytes or 6 h postwounding. Values are shown as normalized read counts. One-way ANOVA Tukey’s multiple comparisons ∗∗∗∗*p* < 0.0001 (n = 5). *E*, immunoblot measurement of CD98 transporter protein levels (SLC3A2 and SLC7A11) in 6 h HDW WT and GCN2KO cell lysates. Fold change in the expression of each protein for this blot is shown, following normalization to GAPDH expression. Representative of three biological replicates.

Given the canonical role for GCN2 as a monitor of amino acid availability *via* uncharged tRNAs (37), we explored whether amino acid deprivation coincided with activation of GCN2 following wounding. Using capillary electrophoresis mass spectrometry to profile amino acid levels during HDW, we observed that free cysteine levels in cells decreased with wounding and were further reduced in GCN2KO cells when translation is not appropriately regulated (Figs. 4*A* and S4). Reduced cysteine upon wounding was also corroborated by the analysis of aminoacylated tRNA^Cys^ in WT and GCN2KO cells following wounding. There were increased levels of uncharged tRNA^Cys^ in WT cells subjected to wounding, with elevated levels in both unwounded and wounded GCN2KO cells (Fig. 4, *B* and *C*). These results suggest that wounding can enhance levels of uncharged tRNAs that can activate GCN2.

We next addressed the roles that GCN2 plays in ensuring appropriate cysteine levels in wounded keratinocytes. First as noted above, there is elevated protein synthesis in GCN2KO that was exacerbated upon wounding, suggesting that loss of appropriate translation dampening upon eIF2α-P can lead to increased demand for free amino acids (Fig. 2*C*). Furthermore, as noted in the transcriptome analyses (Fig. 3*G*), expression of *SLC3A2* and *SLC7A11* mRNAs, encoding subunits of a cystine/glutamate antiporter system, were increased in WT NTERT cells upon wounding and in GCN2KO cells independent of wounding (Fig. 4*D*). However, levels of SLC3A2 protein were lowered in GCN2KO cells independent of wounding, and SLC7A11 was partly reduced in wounded in the knockout cells (Fig. 4*E*). These results suggest that GCN2 serves to enhance synthesis of SLC3A2 and SLC7A11 proteins, which would ensure appropriate uptake of cystine. Deletion of GCN2 would reduce the levels of these transporter proteins despite elevated gene transcript levels.

### GCN2, generation of ROS, and cytoskeletal dynamics

**Figure 5:**
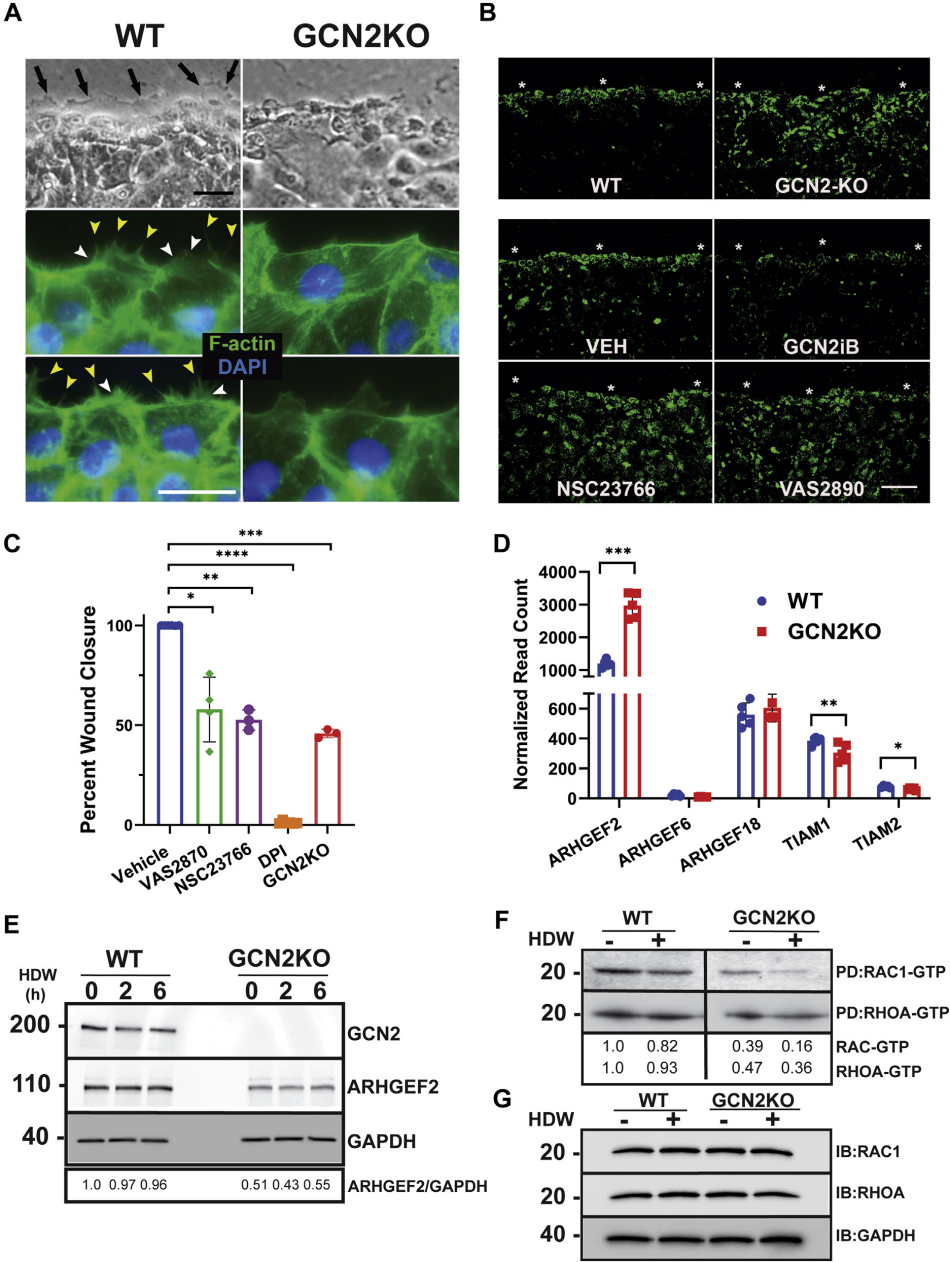
**Loss of reactive oxygen species at the leading edge of wounded GCN2KO keratinocytes is coincident with reduced RAC1-GTP and branching F-actin.***A*, leading edge of migrating keratinocytes at 6 h postwounding in both WT and GCN2KO cells. *Top two panels* demonstrate ruffled edge (*black arrows*) of WT keratinocytes that are absent in GCN2KO keratinocytes (phase contrast, *black bar* = 20 μm). *Bottom four panels* stained with phalloidin-FITC (F-actin, *green*) and DAPI (nuclei, *blue*). Filopodia (*yellow arrows*) and lamellipodia (*white arrows*) are seen on the leading edge keratinocytes of WT but not GCN2KO cells (*white bar* = 10 μm). *B*, ROS production in WT and GCN2KO NTERT keratinocytes subjected to HDW (*top two panels*). In the *lower four panels*, WT NTERT cells were treated with vehicle (DMSO), 5 μM GCN2iB, 5 μM NOX inhibitor VAS2870, or 50 μM RAC1-GTP inhibitor NSC23766, as indicated, and cells were stained for ROS with CellRox Green 6 h after wounding. Cells were imaged with an Opera Phenix confocal microscope (100 μM scale bar). *C*, WT NTERT cells were treated with NOX inhibitors 5 μM VAS2870 or 300 nM DPI, or RAC1-GTP inhibitor 50 μM NSC23766 as indicated, at the time of wounding. As a control, GCN2KO cells were wounded without treatment. Percent wound closure was measured at 16 h post wounding. One-way ANOVA Dunnet’s multiple comparisons, (n = 3). *D*, levels of RAC1-GTP exchange factors *ARHGEF2*, *ARHGEF6*, *ARHGEF18*, *TIAM1*, *and TIAM2* mRNAs in WT *versus* GCN2KO cells. Values are presented as normalized read counts from our RNA-seq analyses. Multiple unpaired t-tests, (n = 5). *E*, WT and GCNKO NTERT cells were subjected to wounding for up to 6 h and ARHGEF2, GCN2, and GAPDH levels were measured by immunoblot analyses. *F*, measurements of RHOA-GTP and RAC1-GTP by affinity purification assays. GST-Rhotekin or GST-PAK1-PBD fusion protein was used to bind the activated form of GTP-bound RHO or RAC1, respectively, in equivalent amounts of lysates prepared from WT and GCN2KO NTERT cells subjected to HDW (+) or no wounding (−). Levels of bound activated RHOA and RAC1 were collected using glutathione beads then measured by immunoblot using an antibody specific to total RAC1 or RHOA proteins. The measurements for RAC1-GTP and RHOA-GTP in the WT and GCN2KO cells were carried out in the same in same immunoblot experiment and therefore are directly comparable between the two panels. Quantification is provided below the panels and is normalized to WT not subject to wounding. *G*, measurements of total RAC1, RHOA, and GAPDH proteins from the lysates prepared from WT and GCN2KO NTERT cells subjected to HDW (+) or no wounding (−). Error bars are SD. ∗*p* < 0.05, ∗∗*p* < 0.01, ∗∗∗*p* < 0.001, and ∗∗∗∗ represents *p* < 0.0001.

Keratinocytes proximal to the wound undergo a transformation of the actin cytoskeleton that expands into the leading edge membrane, creating a directional force to propel the keratinocytes forward. Lamellipodia and filopodia are specialized F-actin-containing structures visible on the leading edge keratinocytes in a scratch wound. Lamellipodia have been described as a ruffled border (5), which is clearly visible extending from KCCM of the WT cells (Fig. 5*A*, black arrows). By comparison, no ruffled border is visible at the front of KCCM in GCN2KO cells. When the keratinocytes were stained with phalloidin-FITC to visualize F-actin bundles, WT keratinocytes displayed numerous fine F-actin-containing filopodia (yellow arrows) and lamellipodia (white arrows) in the leading edge keratinocytes of WT (Fig. 5*A*). Once again, these F-actin structures were largely absent in GCN2KO cells.

Given that balanced production of ROS at the leading edge is a key event in the cytoskeletal reorganization in KCCM (12, 13, 38) and GCN2KO altered expression of genes involved in ROS production (Fig. 4, *E* and *H*), we next stained WT and GCN2KO keratinocytes with CellROX green, which emits fluorescence in the presence of oxidative conditions (39). CellROX green staining revealed organized ROS accumulation predominantly localized to cells within the leading edge of WT wounded cells (Fig. 5*B*). However, ROS staining in the GCN2KO cells was not appropriately situated in cells at the leading edge of the wound but instead there was disorganized fluorescence in cells scattered throughout the keratinocyte monolayer. Furthermore, pharmacological inhibition of GCN2 using GCN2iB in WT cells subject to wounding showed a KCCM with impaired localized ROS production (Fig. 5*B*).

RAC1 activation of NOX at the leading edge of wounded cells has been shown to be critical for localized ROS production (38) and our RNA-seq analysis suggested that RAC1 signaling was disrupted in GCN2KO keratinocytes (Fig. 4, *E* and *H*). To directly determine whether inhibition of RAC1 activation or direct inhibition of NOX disrupts ROS production at the leading edge and the potential contribution of GCN2 in this signaling process, wounded keratinocyte monolayers were treated with small-molecule inhibitors targeting RAC1 activation (NSC23766) or NOX (VAS2870, DPI) and stained with CellROX green. Application of each of these inhibitors impaired the coordinated production of ROS at the leading edge (Fig. 5*B*), similar to what was seen in GCN2KO cells. Furthermore, the pharmacological inhibition of RAC1 and NOX reduced the wound closure rate of WT NTERT cells to levels observed with loss of GCN2 (Fig. 5*C*).

Cellular activators of RAC1-GTP include the GTP exchange factors (GEFs) ARHGEF2, ARHGEF6, ARHGEF18, TIAM1, and TIAM2. Expression of *ARHGEF2* mRNA as judged by RPM reads in our RNA-seq analysis was the highest among these GEFs and was further enhanced upon knockout of GCN2 (Fig. 5*D*). By comparison, *TIAM1* and *TIAM2* transcripts levels were modestly, but significantly, decreased in GCN2KO cells. No change in *ARHGEF6* or *ARHGEF18* expression was detected between WT and GCN2KO keratinocytes. While the *ARHGEF2* mRNA levels were enhanced in GCN2KO cells, the levels of ARHGEF2 protein were lower with loss of GCN2, suggesting GCN2 functions to enhance the synthesis of ARHGEF2 (Fig. 5*E*). ARHGEF2 is known to activate both RAC1 and RHOA for lamellipodia expansion into the leading edge (40, 41, 42).

To more directly determine whether loss of GCN2 disrupts activation of RAC1 and RHOA, we used an affinity purification system that bound active GTP forms of both proteins. Immunoblot analyses of active RAC1 or active RHOA from equal input lysates from WT or GCN2KO cells showed that GCN2 is required to maintain their fully active forms in an unwounded monolayer of keratinocytes and during wounding (Fig. 5, *F* and *G*). RAC1-GTP was reduced in unwounded GCN2KO, with further diminishment upon wounding. RHOA-GTP levels were lowered about twofold in GCN2KO independent of wounding. There were no significant differences in the levels of total RAC1 and RHOA proteins between wounded or unwounded WT and GCN2KO cells. Taken together, these results suggest an important role for GCN2 in induction of ROS at the leading edge *via* RAC1 activation of NOX or RHOA activation to drive the subsequent expansion of the leading edge actin structures to facilitate KCCM.

### Role for GCN2 in an *in vivo* model of wound healing

**Figure 6:**
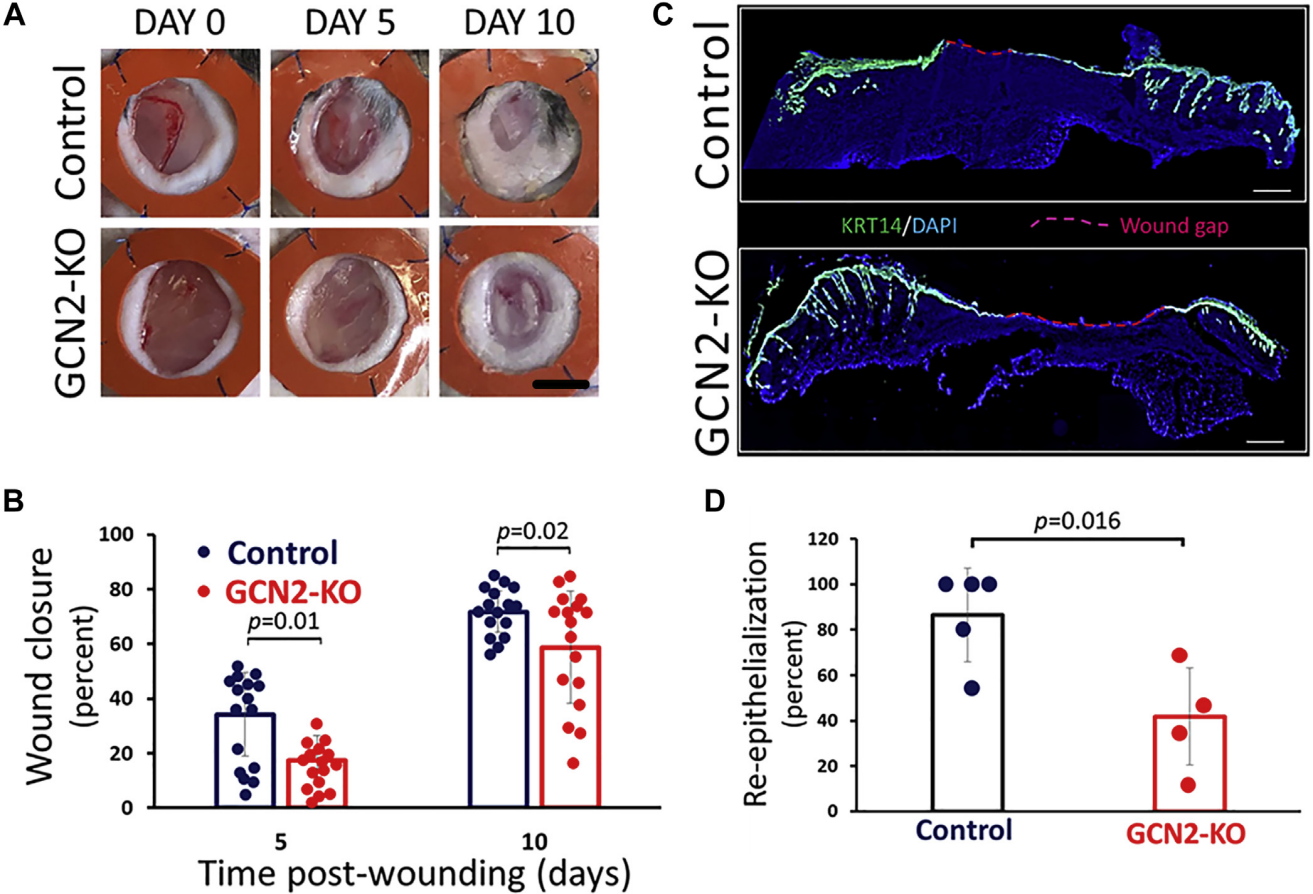
***In vivo* wound healing is impaired in the absence of GCN2.** Circular excisional wounds (8 mm) were created on each flank of WT (*blue dots*) and GCN2KO (*red dots*) C57BL/6J mice. Silicone splint donuts were placed over the opening of each wound to ensure wound closure occurred due to epithelial sheet migration. *A*, digital images of wounds from control and GCN2KO mice day on Day 0, Day 5, and Day 10 postwounding were captured. Representative images for the experiment are provided. The *black scale bar* is 5 mm. *B*, the percent wound closure at Day 5 and Day 10 was determined following comparison to the wound opening at Day 0. Each *dot* on the graph represents an individual measurement; the *solid bars* indicate the mean, and the *dotted boxes* represent the SD for each set. Indicated *p*-values were determined by two-tailed student *t* test. *C*, at Day 10, the splints were removed, the wounded skin harvested, and cryosections spanning the wound bed were created. To identify the location of migrating sheets of epidermis, the cryosections were stained with an antibody to KRT14 (*green*) and with DAPI (*blue*) to identify cellular nuclei as visualized by microscopy. The remaining wound gap (*red*) was determined by measuring the total wound distance and subtracting the distance the epithelial sheets had migrated. The scale bar is 500 μm. *D*, graphical representation of the percentage of re-epithelialization by measuring the remaining wound gap at Day 10, as described in (*C*). Each *dot* on the graph represents an individual measurement; the *solid bars* indicate the mean, and the *dotted boxes* represent the SD for each set. Indicated *p*-values were determined by two-tailed student *t* test.

While a wealth of information can be obtained from studies using two-dimensional cell culture, it is important to validate the key results using three-dimensional skin *in vivo*. Therefore, we compared the wound healing capabilities of murine skin using C57BL/6J mice and C57BL/6J mice containing a homogeneous deletion of GCN2 (GCN2KO; *Eif2ak4*^*tm1.2Dron*^/J). A splinted excisional wound model was used to deter muscle contraction of the wound edge (43, 44). This approach is necessary to mimic the human mode of wound healing that involves re-epithelization and formation of granulation tissue instead of the wound margin contracture used by rodents (45). Quantification of the wound size at both day 5 and 10 post wounding showed that wound closure was significantly impaired in GCN2KO animals compared with the WT (Fig. 6, *A* and *B*). Furthermore, at day 10, the wounded skin was removed and the gap was determined in the cryosections spanning the wound bed that were stained with an antibody to KRT14 and with DAPI to identify cellular nuclei and visualized by microscopy (Fig. 6, *C* and *D*). These experiments also showed that there was significant reduction in wound re-epithelialization in the GCN2KO skin compared with WT. These results support an essential role for GCN2 to facilitate wound closure in the context of full thickness skin.

## Discussion

**Figure 7:**
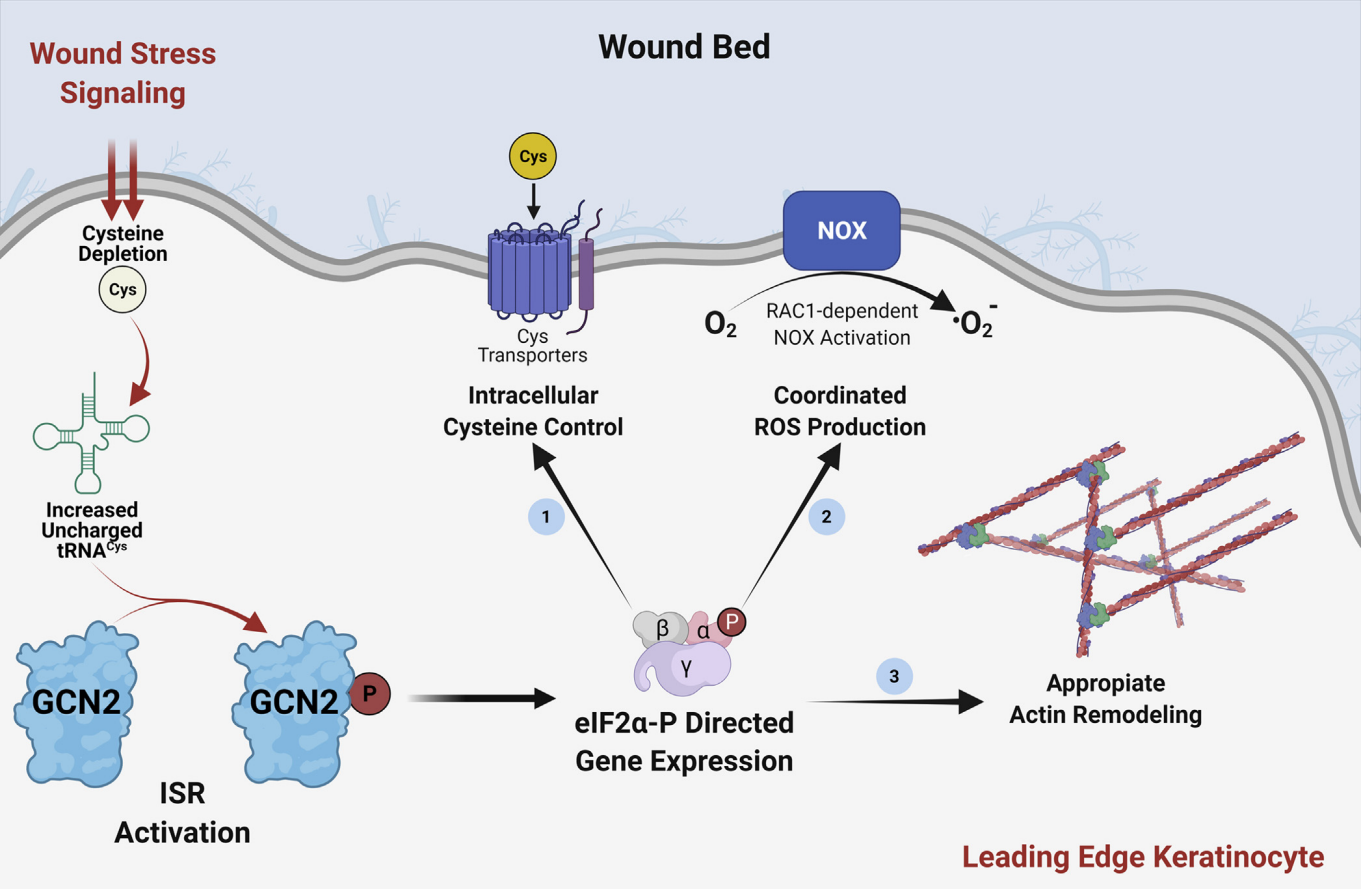
**Model for the role the activation and function of GCN2 in KCCM and wound healing.** The wound bed is illustrated, along with the leading edge of keratinocytes. Wound stress signals activation of GCN2, which is indicated by its phosphorylation. The induced GCN2 serves to maintain elevated levels of phosphorylated eIF2α and attendant translational control. With wounding of keratinocytes, there is a reduction of select free amino acids, including cysteine levels that culminate in lowered aminoacylation of tRNA^Cys^, which is suggested to be a direct activator of GCN2. Lowered cysteine levels in response to wounding are suggested to be a consequence of the demands of translation and the role of cysteine in glutathione production and ROS management. Induced GCN2 facilitates KCCM during wounding by multiple mechanisms: (1) enhanced synthesis of cystine transporters, including SLC7A11 and SLC3A2, that serves to maintain cysteine levels; (2) increased RAC-dependent ROS production by NOX enzymes; and (3) through appropriate actin remodeling. Availability of intracellular cysteine is important for glutathione production and management of ROS; and ROS generation and localization at the leading edge are important for appropriate remodeling of the cytoskeleton.

Herein we present the discovery that GCN2 eIF2α kinase is critical for KCCM and wound closure. There is elevated eIF2α-P in differentiated keratinocytes, and activation of GCN2 is essential for maintaining eIF2α-P upon wounding, whereas deletion of GCN2 led to sharply lowered levels of eIF2α-P and enhanced bulk protein synthesis by 4 h post wounding (Fig. 2, *B* and *C*). As highlighted in the model illustrated in Figure 7, induced GCN2 and attendant gene expression direct key processes that are critical for KCCM. With wounding of keratinocytes, there is a reduction of select free amino acids including cysteine (Figs. S3 and S4*A*), which is suggested to be an activator of GCN2. Induced GCN2 provides for maintenance of cysteine levels, which are important for appropriate protein synthesis and control of ROS, both processes critical for KCCM. Maintenance of cysteine levels is also suggested to be critical for activation of GCN2 *via* tRNA^Cys^ charging that are diminished upon wounding (Fig. 3*B*). Furthermore, GCN2 is important for F-actin remodeling, along with the generation and localization of ROS, in the leading edge on wounded keratinocytes. While the ruffled border was readily detected in wounded WT keratinocytes, these leading-edge structures were absent in GCN2KO cells (Fig. 5*A*). The F-actin bundles extending in the migrating sheet of keratinocytes were largely absent in the GCN2-deficient cells. Appropriate ROS production is critical for cytoskeletal reorganization in the KCCM, and while WT cells showed ROS situated at the leading edge, the genetic or pharmacological disruption of GCN2 led to diminished and disorganized ROS (Fig. 5*B*). Finally, the importance of GCN2 in wounding healing was confirmed using splinted excisional wound model in mice. Mice deleted for GCN2 showed a significant impaired in the closure of wound (Fig. 6) Together, these results indicate that GCN2 contributes to KCCM by multiple processes, and the loss of GCN2 in keratinocytes impairs many key processes that are critical for implementing wound healing (Fig. 7).

### GCN2 directs an ISR that can dispense ATF4 function for KCCM and wound healing

It was surprising that while an induced expression of ATF4 protein occurs in response to wound healing, this process occurred independent of GCN2 (Fig. 2*B*). Furthermore, we showed that ATF4 is dispensable for the GCN2-directed KCCM and wound healing (Fig. S2). These results suggest that the ISR in wound healing involves other ISR target genes that contribute to critical structural and metabolic processes in the KCCM. We previously reported that the ISR functioned independent of ATF4 through other target genes in response to UV irradiation and differentiation in keratinocytes (17, 18, 20). For wound healing, our RNA-seq and biochemical analyses suggested that GCN2 contributes to activation of RAC1 and RHOA through a process that involved regulation of ARHGEF2 expression (Fig. 4, *D*–*F*). While *ARHGEF2* mRNA was significantly enhanced in GCN2KO cells, ARHGEF2 protein was diminished (Fig. 5*E*). These results suggest that GCN2 contributes to increased translation of *ARHGEF2*, which would enhance the GDP to GTP exchange for RAC1 and RHOA that would contribute to the actin remodeling in KCCM (Fig. 5, *F* and *G*). Furthermore, GCN2 enhanced the expression of cystine transporters, SLC3A2 and SLC7A11, supporting the idea that the eIF2α kinase has a critical function in ensuring cysteine levels in keratinocytes. It is noteworthy that despite the reductions in protein levels, *ARHGEF2*, *SLC3A2*, and *SLC7A11* transcript levels are each elevated in GCN2KO cells (Figs. 4*D* and 5*D*). These results suggest that eIF2α-P by GCN2 likely plays a critical role in their enhanced mRNA translation. Other mechanisms independent of the ISR are suggested to contribute to increased transcript levels for these genes, but without GCN2, these transcriptome changes are insufficient to signal enhanced ARHGEF2, SLC3A2, and SLC7A11 protein levels.

The eIF2α kinase PERK monitors the integrity of the endoplasmic reticulum and accompanying secretory pathway (14, 15, 46), which is important preparation of the ECM that is integral for KCCM (8). Our preliminary studies suggest that deletion of PERK in keratinocytes can disrupt proper ECM and keratinocyte differentiation. These results suggest that multiple eIF2α kinases in the ISR can function in the maintenance of the health of skin.

### Therapeutic implications of the ISR in wound healing

Chronic wounds are associated with diabetes, aging, and poor nutrition (1, 2, 3), necessitating complex and long-term care. The ISR is typically associated with resolution of acute stresses. However, with chronic environmental and physiological stresses, the ISR can be altered from its adaptive functions to one that signals significant tissue damage (15, 19, 47). Given the important functions of GCN2 in the KCCM and wound healing, it is inviting to suggest that aberrant regulation of the ISR is a contributor to the transitions to chronic wounds. Many of the underlying contributors to chronic wounds potently modify the ISR. For example, hyperglycemia and poor nutrition invoke eIF2α-P and translational control. In these conditions, the multiple stresses may shift the ISR from its functioning adaptive window to one that is hyperactivated and maladaptive. Furthermore, deficiencies in GCN2 functions due to genetic alterations and aging may impair appropriate ISR and deter the KCCM. Therefore, the ISR and translational control may be a critical therapeutic target to improve wound healing outcome. There are a number of small molecules reported that contribute to enhanced or inhibition of the eIF2α-P and translational control (15, 33, 48), and future studies should investigate the utility of these and related drugs for treatment of chronic wounds.

## Experimental procedures

### Cell culture

Experiments were either performed using NTERT, an immortalized human keratinocyte cell line that has been shown to have normal keratinocyte differentiation properties (49), or normal human keratinocytes (NHK) that were isolated from foreskin tissue as previously described (25) and with methods approved by the Indiana University School of Medicine Institutional Review Board. Both NTERT and NHK cells were maintained and passaged in low calcium EpiLife Complete media (Thermo Fisher Scientific) supplemented with human keratinocyte growth supplement (HKGS; ThermoFisher Scientific) and 1000 U Penicillin-Streptomycin (PS) (Gibco Laboratories). Human keratinocytes are maintained in a basal undifferentiated state by controlling the amount of calcium and fetal bovine serum (FBS) in supplemented media. If calcium and FBS concentrations are increased to 2 mM and 2%, respectively, the NTERT cells respond by inducing an appropriate program of differentiation (50). We previously reported that loss of GCN2 can delay keratinocyte differentiation, but by 72 h this process is implemented (20). Keratinocytes were treated with different stress agents, as indicated, in complete EpiLife HKGS supplemented media either during differentiation into a monolayer or during the wounding process. Media supplements included 1 μM guanabenz acetate (R and D Systems), 5 μM GCN2iB (MedChemExpress), 1 μM cystine (Sigma), 50 μM NSC23766 (Tocris), 5 μM VAS2870 (Tocris), 300 nM DPI (Tocris), and 1 μM puromycin (Calbiochem).

### CRISPR

To create a pool of knockout keratinocytes, Alt-R CRISPR-Cas9 crRNAs were designed with the Integrated DNA Technologies Custom Alt-R CRISPR-Cas9 guide RNA design tool to target the kinase domain of GCN2 either in exon 2 AltR1/UUGUACCCUCAAGGCCUAACGUUUUAGAGCUAUGCU/AltR2 or in exon 12 AltR1/UUGUACCCUCAAGGCCUAACGUUUUAGAGCUAUGCU/AltR2. Early passage GCN2KO cells (six passages or less) were used in all experiments. To knock out *ATF4*, two guides were designed and used in combination to target and flank an 808 base pair sequence in exon 4. The first *ATF4* guide sequence was AltR1/GGAUUUGAAGGAGUUCGACUGUUUUAGAGCUAUGCU/AltR2, and the second was AltR1/GCUCCUGACUAUCCUCAACUGUUUUAGAGCUAUGCU/AltR2. The crRNA and Alt-R CRISPR-Cas9 tracrRNA were prepared in equimolar concentrations to a final sgRNA duplex concentration of 100 μM and annealed by heating at 95 °C for 5 min and allowing to cool to RT. RNP complexes were formed by incubating the above sgRNA duplex with Alt-R S.p and HiFi Cas9 Nuclease at RT for 20 min. Complexes with exon 2 or exon 12 targeted guide RNAs were transfected by nucleofection using one million keratinocytes using the Amaxa Nucleofector II device (Lonza) with the Amaxa Human Keratinocyte Nucleofector kit (VPD-1002, program T-018) and Alt-R Cas9 Electroporation Enhancer. Successful gene editing was assessed first by using the Surveyor Mutation Detection kit (Integrated DNA Technologies 706020), and then protein knockdown was confirmed by immunoblot analysis of GCN2 and ATF4.

### Collective cell migration and wounding

Two methods were developed to evaluate KCCM during the re-epithelialization process of wounding healing. First, a kinetic and automated 96-well cell wound assay from IncuCyte (ZOOM model) was utilized. This system was comprised of a 96-well WoundMaker Tool (4563), a software module (9600-0012), and ImageLock Plates (4679). The 96 pinheads of the WoundMaker were used to create a single, uniform, 700 to 800 micron wounds in a differentiated keratinocyte monolayer grown on ImageLock 96-well microplates. On day 1, 40,000 keratinocytes per well were plated in Imagelock 96-well plates in normal EpiLife media supplemented with HKGS and allowed to recover overnight and reach confluence. The next day, the keratinocytes were treated with media supplemented with 2% FBS and 2 mM calcium chloride to induce differentiation (50). Keratinocytes begin an early differentiation process (36 h) that transforms the individual cells into a monolayer expressing involucrin and creates cell–cell attachments. This was a critical time optimized to mimic re-epithelialization of collectively migrating basal and suprabasal keratinocytes, but not to terminally differentiate and lose the capacity for CCM (5). At 36 h post treatment with FBS and calcium, the media was removed from the cells and replaced with HBSS. The ImageLock plate was then transferred to the WoundMaker Tool and subjected to a uniform wound. Any debris and loose cells were washed away with HBSS containing Ca^2+^ and Mg^2+^. The growth media was then replaced and imaged for CCM at select time intervals using the Incucyte Zoom imager.

The second method that was developed to evaluate KCCM and wound healing maximized the proportion of wounded leading-edge keratinocytes to enhance measurements of the biochemical and molecular changes that drive collective cell migration. On the first day, one million keratinocytes were plated in a 6-well plate and allowed to recover overnight and reach confluence. The next day, the keratinocytes were differentiated by culturing in EpiLife media supplemented with 2% FBS and 2 mM calcium chloride. At 36 h post treatment, multiple uniform wounds were created in the plate wells with seven multichannel pipet tips that were 1 mM wide and spaced 5 mM apart. The pipet tips were drawn across the width of the 6-well plate and then turned 90 degrees and wounded again the same way to create a high-density wounded (HDW) monolayer. After a designated period of CCM, the media was removed from the plate and the cells were washed in cold PBS and lysed for mRNA or protein analyses.

### Immunoblot analyses

Cell lysates were prepared by removing the growth media and washing the cells in culture in cold PBS and aspirating. Cells were either lysed directly in a breaking solution containing 1% SDS and 1× HALT protease and phosphatase inhibitor (Pierce 78429) or in a lysis solution provided in Active RHOA and RAC1 detection kits (Active RAC1 detection kit, CST8815; Active RHOA detection kit, CST8820). Lysates were sonicated for 5 s and heated at 95 °C for 5 min, followed by a brief clearance by centrifugation at 10,000*g*. Protein concentrations were determined by the BCA assay (Pierce). Equal amounts of the protein preparations (ranging from 5 to 20 μg of lysate depending on targeted protein and applied antibody) were prepared with 6× Tris-HCL loading dye (Boston Bioproducts) and 10× denaturing solution (Invitrogen) for a total of 30 μl. Samples were heated at 95 °C for 5 min, and separated electrophoresis in BioRad 4 to 20% Tris-HCL gels with 1× Tris-Glycine/SDS running buffer (BioRad 161-0732). Protein MW ladders used in the gel electrophoresis were prepared by mixing equal volumes of SeeBlue Plus2 prestained protein ladder (Invitrogen LC5925) and Magic Mark XP (Invitrogen LC5602). Electrophoresis was carried out at 50 V for 15 min, then at 125 V until the bromophenol blue dye front reached the bottom of the gel. Gels were first rinsed in 25% ethanol for 2 to 5 min before being transfer to nitrocellulose (BioRad 170-4159) with the BioRad turbo blotting unit (BioRad 170-4155). Protein bound blots were then blocked in an immunoblot solution containing TBS, 5% milk, and 0.1% tween-20 for 60 min at room temperature with gentle rocking. Blots were probed with the indicated primary antibodies diluted at 1:1000 overnight with rocking in immunoblot solution. The primary antibodies used include: ATF4, Cell Signaling Technology (CST), 11815, clone D4B8; GAPDH, CST 2118, clone 14C10; Actin, CST 4970, clone13E5; IVL, Abcam Ab181980, EPR13054; eIF2α, CST 5324; eIF2α-P, CST3398; puromycin, EMDMillipore, MABE343; CD98, Abcam Ab108300, EPR3548(2); xCT (SLC7A11), Abcam Ab175186, EPR8290(2); GCN2, Abcam Ab134053, EPR5970(2); P-GCN2 Abcam Ab75836, EPR2320Y; GADD34, Proteintech 10449-1-AP; CReP, Proteintech 14634-1-AP; ARHGEF2, Cell Signaling Technology 4076, clone 55B6; RAC1, CST 2465; and Cell Signaling Technology RhoA (67B9) rabbit mAb #211. To remove unbound antibodies, blots were washed four times for 10 to 15 min each at room temperature in TBS solution containing 0.1% tween-20, followed by incubation with the respective HRP-conjugated secondary antibodies for 1 h at room temperature. Blots were further washed four times for 10 to 15 min each at room temperature in a TBS solution supplement 0.1% tween 20, and the targeted proteins were visualized with Pierce SuperSignal West Femto (34095) and imaged on a LAS4010 Luminescent Analyzer. At least three biological replicates were carried out for each immunoblot measurement.

### Measurements of protein synthesis

Protein synthesis was measured by the amount of puromycin incorporated into total cellular protein as described (51). Culture plates with early differentiated (36 h) keratinocyte monolayers were wounded and then incubated at 37 °C for 4 h. WT and GCN2KO ells were treated with HDW wounding for up to 6 h or no stress (0 h). Alternatively, as control WT cells were treated with 1 mM thapsigargin for 6 h. 30 min prior to harvesting, cells were cultured with 1 μM puromycin (Calbiochem). Media was removed and cells were washed with cold PBS solution before lysing for immunoblot measurement as described above. An anti-puromycin antibody (EMD Millipore clone 12D10) was used to detect incorporated puromycin into 10 μg of cellular lysate.

### RNA isolation, real-time PCR, and cDNA synthesis

Total RNA was isolated using RNeasy Plus kit according to the manufacturer’s instructions (Qiagen). Keratinocytes were washed with PBS and lysed directly in the plate with RLT buffer. RNA was measured with a Nanodrop and 260/280 ratios determined. FAST Advanced RT and TaqMan Fast Advanced Master Mix (Thermo Fisher) were used to prepare cDNA using 1 μg of total RNA in a 20 μl reaction. Gene targets were amplified with FAM-labeled gene expression assays from Thermo Fisher (*ATF4* Hs00909569_g1 Thermo Fisher 4331182; *GCN2* Hs01010957_m1, Thermo Fisher 4331182; *GAPDH* Hs02786624_g, Thermo Fisher 4331182). Real-time PCR reactions were performed on a QuantStudio 7 Flex Real-Time PCR System (Thermo Fisher Scientific) using FAST Advanced QPCR defined incubation temperatures and times for 40 cycles. The delta–delta CT method was used to calculate relative changes in expression values between a reference housekeeping gene (*GAPDH*) and genes of interest.

### RNA preparation and sequencing

Human keratinocyte WT and GCN2KO NTERT cells were plated and left unwounded or subjected to HDW in replicates of five. Post 6 h wounding, total RNA was extracted from unwounded and HDW WT and GCN2 KO NTERTs cells using RNA easy plus kit (Qiagen) according to the manufacturer’s instructions. Total RNA was submitted to GENEWIZ where they performed library preparation and sequencing. A total of 20 samples were submitted to GENEWIZ, which included five replicates each for WT NTERTs unwounded and HDW and GCN2 KO NTERTs unwounded and HDW. Illumina HiSeq platform was used for sequencing with 150 bp paired-end reads. GENEWIZ performed differential expression (DE) analysis from raw RNA sequencing data with their standard pipeline and provided us with differentially expressed genes (DEG) list among different groups. The analysis pipeline is mentioned in brief below. After the quality check, the raw sequencing reads were trimmed for possible adapter sequences and nucleotides with poor quality using Trimmomatic v.0.36 (52). The trimmed sequencing reads were aligned to human reference genome GRCh38 available on ENSEMBL using a STAR aligner v.2.5.2 b (53). Out of total 686,088,882 read pairs, 610,544,244 mapped uniquely to the human genome (89%). The number of uniquely mapped reads to the exons of different genes was counted using a program called featureCounts from the Subread package v.1.5.2 (54). Lastly, the package DESeq2 was used to determine differentially expressed genes among different groups (35). First, we compared WT unwounded samples and GCN2 KO unwounded samples for DE analysis. The second group comparison for DE analysis was between WT HDW samples and GCN2 KO HDW samples. For the third and fourth group DE analysis, WT unwounded and GCN2 KO unwounded were compared with WT wounded and GCN2 KO wounded, respectively. The genes with log2fold change of ≥ ±1 and *p*-adjusted value of ≤0.05 were considered significant for further analyses. These genes were used for performing pathway enrichment analysis using IPA (Qiagen). RNA-seq datasets from this study are available in the NCBI GEO database (accession # GSE171666 with the private token number for reviewers: gbqhmeyartizlgz).

### Bioinformatic analysis

To determine which biological pathways were significantly regulated in HDW datasets of differentially regulated transcripts, DE gene datasets were submitted for IPA core analysis (Ingenuity Pathway Analysis version 60467501, Qiagen). For each biological function assigned by IPA, a statistical quantity called the activation z-score is calculated. The score is used to predict the probability of a biological function being in an active state. To identify differences in regulated biological pathways between treatment groups, the core analysis was followed by a comparison analysis in IPA. Finally, the Upstream Regulator tool was used to determine the biological impact of upstream molecules according to the genes they regulate. The Upstream Regulator tool assigned an activation z-score for that gene network (55).

### ZipChip capillary electrophoresis mass spectrometry

The ZipChip CE ion source from 908 Devices was interfaced with a Thermo Fisher LTQ velos Orbitrap MS (Thermo). Microfluidic chips with a 10 cm separation channel (HS, 908 Devices Inc) and the Metabolite Assay Kit (908 Devices Inc) were used for measurement of intracellular free amino acids. An injection volume of 4 nl was used, and the separation was run at a field strength of 1000 V/cm. A calibration curve was prepared using a sample containing all 20 amino acids (Promega L4461). The amino acid standard was diluted 1:100 to make 10 μM standard in BGE metabolite diluent provided in the 908 device kit. A four-point calibration curve was made by diluting the 10 μM standard 1:2. The mass spectrometer was run in positive, profile mode scanning from 70 to 500 m/z in the LTQ orbitrap. Runtime was 4 min. The exact M + H was calculated for each amino acid to construct a configuration file. MZMine2 software (56) was then used to calculate the area under the curve for each amino acid peak. Each area was normalized to another amino acid within the same sample lysate/run. Cell lysates for CE/MS were prepared from two million keratinocytes. Monolayers were trypsinized and harvested by centrifugation, washed in 1× cold PBS, carefully aspirated to remove residual liquid. Cells were lysed in 50 μl water containing 20% HPLC grade methanol and 0.1% methylmercaptoethanol as an antioxidant. The lysate was spun down to pellet cell debris and transferred to a new tube. Lysates were prepared and analyzed immediately to avoid changes in cysteine or tryptophan due to oxidation or light exposure. Ten microliters of lysate was diluted 1:10 in 908 devices provided with metabolite diluent, and 10 μl of diluted lysate was loaded onto the zip chip for separation *via* capillary electrophoresis. The sample separates based on size and charge through the Zipchip, and the sample was electrosprayed from the ZipChip into the MS. If the peaks were of out of scale, the sample was diluted 1:10 again to ensure analysis of samples was in the linear range of the amino acid standards.

### tRNA charging assay

Cellular charged tRNA levels were measured as previously described (57). Briefly, RNA was extracted from cells using TRIzol (Life Technologies, 15596018). RNA was then treated with either 12.5 mM NaIO4 (oxidized) or 12.5 mM NaCl (nonoxidized control) in sodium acetate buffer (pH = 4.5) in dark at room temperature for 20 min, followed by quenching with 0.3 M glucose. Samples were spiked with 7.3 ng yeast tRNA^Phe^ (R4018, Sigma) and then subjected to desalination using a MicroSpin G-50 column (27533001, GE Healthcare). Next, RNA was deacetylated (tRNA discharging) in 50 mM Tris-HCl (pH = 9.0) solution at 37 °C for 45 min. Following deacylation, 5′-adenylated adaptor (5′-/5rApp/TGGAATTCTCGGGTGCCAAGG/3ddC/-3′) DNA oligomer was ligated to the tRNA using T4 RNA ligase2 truncated KQ (M0351L, New England BioLabs). An oligomer (5′-GCCTTGGCACCCGAGAATTCCA-3′), complementary to the adaptor sequence, was used for cDNA synthesis using SuperScript IV RT kit (Invitrogen, 18090050). cDNA was used for qPCR-based detection of tRNA with the following primers: yeast phenylalanine fw-5′-GCGGAYTTAGCTCAGTTGGGAGAG-3′, rev-5′-GAGAATTCCATGGTGCGAAYTCTGT GG-3′; human cysteine fw-5′-GGGGGTATAGCTCA-3′, rev-5′-GAGAATTCCATGGAGGG GGCACC-3′; human tryptophan, fw-5′-GACCTCGTGGCGCA-3′, rev-5′-GAGAATTCCATG GTGACCCCGACGTGA-3′. Results were first normalized to yeast phenylalanine tRNA; uncharged tRNA fractions were calculated by subtracting the charged fraction (NaIO4 treated) from total (NaCl treated).

### Phase contrast, fluorescent, and confocal microscopy

A Leica microscope with Intensilight epifluorescence and Qimaging camera were used for phase and fluorescent imaging purposes. Images were taken using a 20× objective lens at 25 °C. Qimaging and Nikon Elements software were used for data acquisition. CellROX Green Reagent is a fluorescent dye (Thermo Fisher) for detecting oxidative stress in living cells. The dye is cell-permeable and does not fluoresce in a reducing environment. When ROS is present, the oxidized dye emits bright green fluorescence and binds DNA, with absorption/emission at 485/520 nm. Wounded monolayers were prepared as described above and allowed to begin preparing for collective migration for 6 h. The CellROX reagent was added to the culture media at a final concentration of 5 μM and incubated with the cells for 30 min at 37 °C. Media was removed and cells were washed three times with PBS solution before imaging at 488 nM wavelength with 20× water objective using the PerkinElmer Opera Phenix High Content Screening System. Images were analyzed using the Harmony analysis software. To visualize actin architecture, confluent monolayers of WT or GCN2KO NTERT cells were wounded and then fixed in 4% paraformaldehyde and permeabilized with 0.1% Triton X-100 (Sigma T8532). Cells were then stained with 1 μg/ml DAPI (4′,6-diamido-2-phenylindole dihydrochloride, Sigma-Aldrich D9542) and fluorescein isothiocyanate labeled Phalloidin (Sigma-Aldrich P5282).

### *In vivo* wound healing model

WT C57BL/6J and *Gcn2*^−/−^ C57BL/6J (*Eif2ak4*^*tm1.2Dron*^) mice were reported (58) and were used for assessment of wound healing using the splinted excisional wound model to deter contraction of the wound edge and mimic the human mode of wound healing that involves re-epithelization and formation of granulation tissue instead of wound margin contracture used by rodents (43, 44, 45). Mice were anesthetized, and fur removed to use punch biopsy tools that create regular 8 mM wounds, and the opening was held in place with a silicone splint (43, 44). This prevented the rodent from contracting the muscle around the skin and closing the wound in a manner not connected to re-epithelialization. The wound was covered with semiocclusive dressing (Tegaderm; 3M). Wound closure was documented by imaging beginning on day 0 and continuing every 5 days for up to 10 days, and wound area was determined using Image J software.

### Immunohistochemistry

Immunostaining of KRT14 was performed on cryosections of wound tissue samples using specific antibodies as previously described (PMID27184784). Briefly, 10 mm thick cryosectioned tissue was fixed with cold acetone, blocked with 10% normal goat serum, and incubated with specific antibodies against KRT14 (Biolegend) overnight at 4 °C (PMID27194784, PMID33214614). Signal was visualized by subsequent incubation with fluorescence-tagged secondary antibody (Alexa 488-tagged) followed by counterstaining with DAPI. Wound re-epithelialization was calculated from KRT14 stained sections (PMID27194784) using ZenBlue (Zeiss) software by measuring the original width of the wound (W) and then measuring the portions of the wound that had re-epithelialized (E). Percent re-epithelialized was calculated as: (E/W) × 100 as previously described (59). All animal experiments were approved by the Indiana University Institutional Animal Care and Use Committee, which ensures compliance with the guide for the care and use of laboratory animals published by the NIH.

### Statistical analyses

GraphPad Prism v9.0.1 was used to generate all graphs and perform relevant statistical tests. Statistical comparisons were performed as appropriate for the experimental design using t-tests, one-way or two-way ANOVA. If significance was measured in the ANOVA, multiple comparisons between groups were made using Dunnett’s or Tukey’s post hoc analysis. A significance level (α) was defined as α = 0.05. Individual values were shown in all bar graphs while data variability as the standard deviation (SD) from the mean or standard error of measure (SEM) was shown in error bars for bar graphs or scatter plot line graphs. Significance was drawn on graphs and bar charts and defined with asterisks using the following format: ∗*p* < 0.05. ∗∗*p* < 0.01, ∗∗∗*p* < 0.001, ∗∗∗∗*p* < 0.0001. Numbers of biological replicates are designated as (n) in each figure legend.

## Data availability

The datasets generated and analyzed in this study are included within the manuscript and supplementary information and can be obtained from the corresponding authors upon reasonable request. RNA-seq datasets from this study are deposited in the NCBI GEO database (accession # GSE171666).

## Supporting information

This article contains supporting information.

## Conflict of interest

R. C. W. serves as a scientific advisor to HiberCell. All other authors declare that they have no conflicts of interest with the contents of this article.

## References

[1] Sen C.K. (2019). Human wounds and its burden: An updated compendium of estimates. Adv. Wound Care (New Rochelle).

[2] Frykberg R.G., Banks J. (2015). Challenges in the treatment of chronic wounds. Adv. Wound Care (New Rochelle).

[3] Han G., Ceilley R. (2017). Chronic wound healing: A review of current management and treatments. Adv. Ther..

[4] Reinke J.M., Sorg H. (2012). Wound repair and regeneration. Eur. Surg. Res..

[5] Rousselle P., Braye F., Dayan G. (2019). Re-epithelialization of adult skin wounds: Cellular mechanisms and therapeutic strategies. Adv. Drug Deliv. Rev..

[6] Pastar I., Stojadinovic O., Yin N.C., Ramirez H., Nusbaum A.G., Sawaya A., Patel S.B., Khalid L., Isseroff R.R., Tomic-Canic M. (2014). Epithelialization in wound healing: A comprehensive review. Adv. Wound Care (New Rochelle).

[7] Poujade M., Grasland-Mongrain E., Hertzog A., Jouanneau J., Chavrier P., Ladoux B., Buguin A., Silberzan P. (2007). Collective migration of an epithelial monolayer in response to a model wound. Proc. Natl. Acad. Sci. U. S. A..

[8] Friedl P., Gilmour D. (2009). Collective cell migration in morphogenesis, regeneration and cancer. Nat. Rev. Mol. Cell Biol..

[9] Ilina O., Friedl P. (2009). Mechanisms of collective cell migration at a glance. J. Cell Sci..

[10] Theveneau E., Mayor R. (2013). Collective cell migration of epithelial and mesenchymal cells. Cell. Mol. Life Sci..

[11] Theveneau E., Steventon B., Scarpa E., Garcia S., Trepat X., Streit A., Mayor R. (2013). Chase-and-run between adjacent cell populations promotes directional collective migration. Nat. Cell Biol..

[12] Stanley A., Thompson K., Hynes A., Brakebusch C., Quondamatteo F. (2014). NADPH oxidase complex-derived reactive oxygen species, the actin cytoskeleton, and Rho GTPases in cell migration. Antioxid. Redox Signal..

[13] Ridley A.J. (2011). Life at the leading edge. Cell.

[14] Wek R.C., Jiang H.Y., Anthony T.G. (2006). Coping with stress: eIF2 kinases and translational control. Biochem. Soc. Trans..

[15] Wek R.C. (2018). Role of eIF2alpha kinases in translational control and adaptation to cellular stress. Cold Spring Harb. Perspect. Biol..

[16] Harding H.P., Zhang Y., Zeng H., Novoa I., Lu P.D., Calfon M., Sadri N., Yun C., Popko B., Paules R., Stojdl D.F., Bell J.C., Hettmann T., Leiden J.M., Ron D. (2003). An integrated stress response regulates amino acid metabolism and resistance to oxidative stress. Mol. Cell.

[17] Collier A.E., Spandau D.F., Wek R.C. (2018). Translational control of a human CDKN1A mRNA splice variant regulates the fate of UVB-irradiated human keratinocytes. Mol. Biol. Cell.

[18] Collier A.E., Wek R.C., Spandau D.F. (2015). Translational repression protects human keratinocytes from UVB-induced apoptosis through a discordant eIF2 kinase stress response. J. Invest. Dermatol..

[19] Marciniak S.J., Ron D. (2006). Endoplasmic reticulum stress signaling in disease. Physiol. Rev..

[20] Collier A.E., Wek R.C., Spandau D.F. (2017). Human keratinocyte differentiation requires translational control by the eIF2alpha kinase GCN2. J. Invest. Dermatol..

[21] Lewis D.A., Yi Q., Travers J.B., Spandau D.F. (2008). UVB-induced senescence in human keratinocytes requires a functional insulin-like growth factor-1 receptor and p53. Mol. Biol. Cell.

[22] Serezani A.P., Bozdogan G., Sehra S., Walsh D., Krishnamurthy P., Sierra Potchanant E.A., Nalepa G., Goenka S., Turner M.J., Spandau D.F., Kaplan M.H. (2017). IL-4 impairs wound healing potential in the skin by repressing fibronectin expression. J. Allergy Clin. Immunol..

[23] Grada A., Otero-Vinas M., Prieto-Castrillo F., Obagi Z., Falanga V. (2017). Research techniques made simple: Analysis of collective cell migration using the wound healing assay. J. Invest. Dermatol..

[24] Papini S., Cecchetti D., Campani D., Fitzgerald W., Grivel J.C., Chen S., Margolis L., Revoltella R.P. (2003). Isolation and clonal analysis of human epidermal keratinocyte stem cells in long-term culture. Stem Cells.

[25] Kuhn C., Hurwitz S.A., Kumar M.G., Cotton J., Spandau D.F. (1999). Activation of the insulin-like growth factor-1 receptor promotes the survival of human keratinocytes following ultraviolet B irradiation. Int. J. Cancer.

[26] Park Y., Reyna-Neyra A., Philippe L., Thoreen C.C. (2017). mTORC1 balances cellular amino acid supply with demand for protein synthesis through post-transcriptional control of ATF4. Cell Rep..

[27] Lassot I., Segeral E., Berlioz-Torrent C., Durand H., Groussin L., Hai T., Benarous R., Margottin-Goguet F. (2001). ATF4 degradation relies on a phosphorylation-dependent interaction with the SCF(betaTrCP) ubiquitin ligase. Mol. Cell. Biol..

[28] Milani M., Rzymski T., Mellor H.R., Pike L., Bottini A., Generali D., Harris A.L. (2009). The role of ATF4 stabilization and autophagy in resistance of breast cancer cells treated with Bortezomib. Cancer Res..

[29] Scortegagna M., Kim H., Li J.L., Yao H., Brill L.M., Han J., Lau E., Bowtell D., Haddad G., Kaufman R.J., Ronai Z.A. (2014). Fine tuning of the UPR by the ubiquitin ligases Siah1/2. PLoS Genet..

[30] Rutkowski D.T., Arnold S.M., Miller C.N., Wu J., Li J., Gunnison K.M., Mori K., Sadighi Akha A.A., Raden D., Kaufman R.J. (2006). Adaptation to ER stress is mediated by differential stabilities of pro-survival and pro-apoptotic mRNAs and proteins. PLoS Biol..

[31] Ait Ghezala H., Jolles B., Salhi S., Castrillo K., Carpentier W., Cagnard N., Bruhat A., Fafournoux P., Jean-Jean O. (2012). Translation termination efficiency modulates ATF4 response by regulating ATF4 mRNA translation at 5' short ORFs. Nucleic Acids Res..

[32] Gardner L.B. (2008). Hypoxic inhibition of nonsense-mediated RNA decay regulates gene expression and the integrated stress response. Mol. Cell. Biol..

[33] Nakamura A., Nambu T., Ebara S., Hasegawa Y., Toyoshima K., Tsuchiya Y., Tomita D., Fujimoto J., Kurasawa O., Takahara C., Ando A., Nishigaki R., Satomi Y., Hata A., Hara T. (2018). Inhibition of GCN2 sensitizes ASNS-low cancer cells to asparaginase by disrupting the amino acid response. Proc. Natl. Acad. Sci. U. S. A..

[34] Tsaytler P., Harding H.P., Ron D., Bertolotti A. (2011). Selective inhibition of a regulatory subunit of protein phosphatase 1 restores proteostasis. Science.

[35] Love M.I., Huber W., Anders S. (2014). Moderated estimation of fold change and dispersion for RNA-seq data with DESeq2. Genome Biol..

[36] Koppula P., Zhang Y., Zhuang L., Gan B. (2018). Amino acid transporter SLC7A11/xCT at the crossroads of regulating redox homeostasis and nutrient dependency of cancer. Cancer Commun. (Lond.).

[37] Baird T.D., Wek R.C. (2012). Eukaryotic initiation factor 2 phosphorylation and translational control in metabolism. Adv. Nutr..

[38] Ushio-Fukai M. (2006). Localizing NADPH oxidase-derived ROS. Sci. STKE.

[39] Choi H., Yang Z., Weisshaar J.C. (2015). Single-cell, real-time detection of oxidative stress induced in Escherichia coli by the antimicrobial peptide CM15. Proc. Natl. Acad. Sci. U. S. A..

[40] Machacek M., Hodgson L., Welch C., Elliott H., Pertz O., Nalbant P., Abell A., Johnson G.L., Hahn K.M., Danuser G. (2009). Coordination of Rho GTPase activities during cell protrusion. Nature.

[41] Nalbant P., Chang Y.C., Birkenfeld J., Chang Z.F., Bokoch G.M. (2009). Guanine nucleotide exchange factor-H1 regulates cell migration *via* localized activation of RhoA at the leading edge. Mol. Biol. Cell.

[42] Lawson C.D., Ridley A.J. (2018). Rho GTPase signaling complexes in cell migration and invasion. J. Cell Biol..

[43] Das A., Ghatak S., Sinha M., Chaffee S., Ahmed N.S., Parinandi N.L., Wohleb E.S., Sheridan J.F., Sen C.K., Roy S. (2016). Correction of MFG-E8 resolves inflammation and promotes cutaneous wound healing in diabetes. J. Immunol..

[44] Ganesh K., Das A., Dickerson R., Khanna S., Parinandi N.L., Gordillo G.M., Sen C.K., Roy S. (2012). Prostaglandin E(2) induces oncostatin M expression in human chronic wound macrophages through Axl receptor tyrosine kinase pathway. J. Immunol..

[45] Chen J.S., Longaker M.T., Gurtner G.C. (2013). Murine models of human wound healing. Methods Mol. Biol..

[46] Walter P., Ron D. (2011). The unfolded protein response: From stress pathway to homeostatic regulation. Science.

[47] Pakos-Zebrucka K., Koryga I., Mnich K., Ljujic M., Samali A., Gorman A.M. (2016). The integrated stress response. EMBO Rep..

[48] Wong Y.L., LeBon L., Basso A.M., Kohlhaas K.L., Nikkel A.L., Robb H.M., Donnelly-Roberts D.L., Prakash J., Swensen A.M., Rubinstein N.D., Krishnan S., McAllister F.E., Haste N.V., O'Brien J.J., Roy M. (2019). eIF2B activator prevents neurological defects caused by a chronic integrated stress response. Elife.

[49] Dickson M.A., Hahn W.C., Ino Y., Ronfard V., Wu J.Y., Weinberg R.A., Louis D.N., Li F.P., Rheinwald J.G. (2000). Human keratinocytes that express hTERT and also bypass a p16(INK4a)-enforced mechanism that limits life span become immortal yet retain normal growth and differentiation characteristics. Mol. Cell. Biol..

[50] Borowiec A.S., Delcourt P., Dewailly E., Bidaux G. (2013). Optimal differentiation of *in vitro* keratinocytes requires multifactorial external control. PLoS One.

[51] Deliu L.P., Ghosh A., Grewal S.S. (2017). Investigation of protein synthesis in Drosophila larvae using puromycin labelling. Biol. Open.

[52] Bolger A.M., Lohse M., Usadel B. (2014). Trimmomatic: A flexible trimmer for Illumina sequence data. Bioinformatics.

[53] Dobin A., Davis C.A., Schlesinger F., Drenkow J., Zaleski C., Jha S., Batut P., Chaisson M., Gingeras T.R. (2013). Star: Ultrafast universal RNA-seq aligner. Bioinformatics.

[54] Liao Y., Smyth G.K., Shi W. (2014). featureCounts: An efficient general purpose program for assigning sequence reads to genomic features. Bioinformatics.

[55] Kramer A., Green J., Pollard J., Tugendreich S. (2014). Causal analysis approaches in ingenuity pathway analysis. Bioinformatics.

[56] Pluskal T., Castillo S., Villar-Briones A., Oresic M. (2010). MZmine 2: Modular framework for processing, visualizing, and analyzing mass spectrometry-based molecular profile data. BMC Bioinformatics.

[57] Jiang J., Srivastava S., Seim G., Pavlova N.N., King B., Zou L., Zhang C., Zhong M., Feng H., Kapur R., Wek R.C., Fan J., Zhang J. (2019). Promoter demethylation of the asparagine synthetase gene is required for ATF4-dependent adaptation to asparagine depletion. J. Biol. Chem..

[58] Maurin A.C., Jousse C., Averous J., Parry L., Bruhat A., Cherasse Y., Zeng H., Zhang Y., Harding H.P., Ron D., Fafournoux P. (2005). The GCN2 kinase biases feeding behavior to maintain amino acid homeostasis in omnivores. Cell Metab..

[59] Roy S., Biswas S., Khanna S., Gordillo G., Bergdall V., Green J., Marsh C.B., Gould L.J., Sen C.K. (2009). Characterization of a preclinical model of chronic ischemic wound. Physiol. Genomics.

