# Supplementary material for: The eIF2 kinase GCN2 directs keratinocyte collective cell migration during wound healing via coordination of reactive oxygen species and amino acids
Source: J Biol Chem. 2021 Sep 29;297(5):101257. doi: 10.1016/j.jbc.2021.101257 (PMC8554533; doi:10.1016/j.jbc.2021.101257)
Supplement: Supplemental Figures S1–S4 [file mmc1.pdf]

SUPPLEMENTARY FIGURES

The eIF2 kinase GCN2 directs keratinocyte collective cell migration during wounding by proper coordination of reactive oxygen species and amino acids

Rebecca R. Miles, Parth H. Amin, Miguel Barriera Diaz, Jagannath Misra, Erica Aukerman, Amitava Das, Nandini Ghosh, Tanner Guith, Michael D. Knierman, Sashwati Roy, Dan F Spandau, and Ronald C. Wek

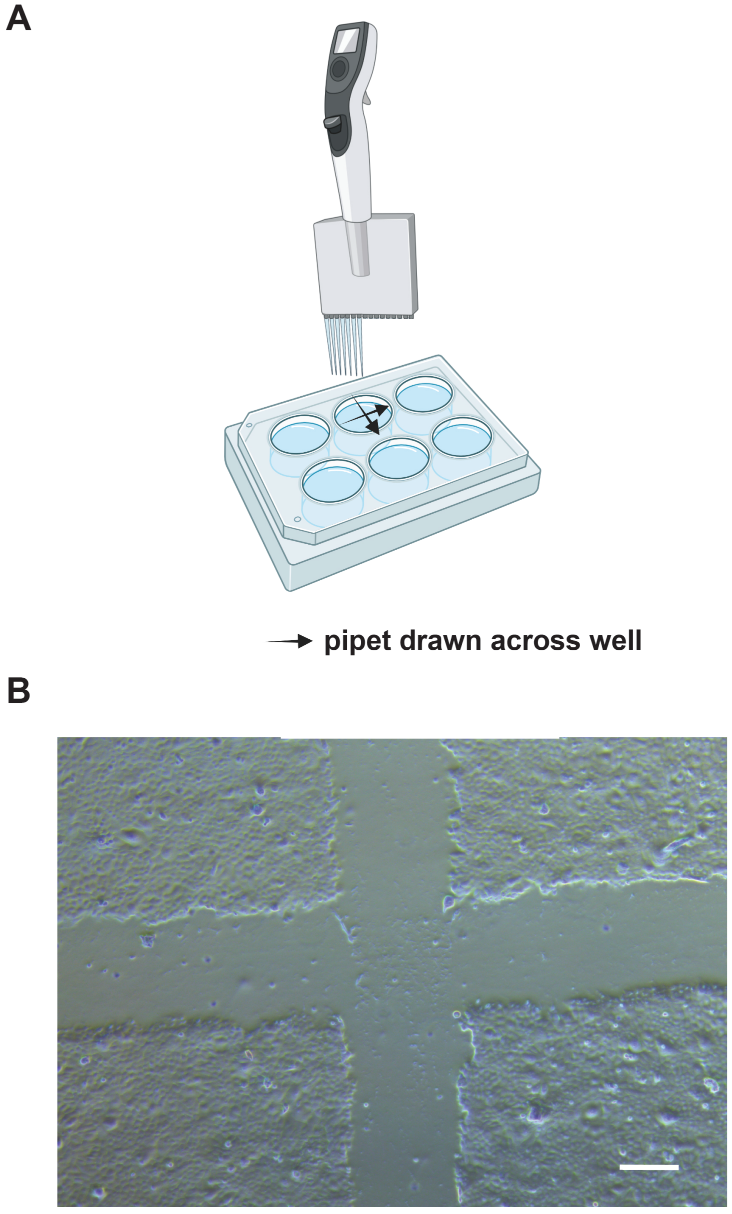

Supplemental Figure 1. Illustration of high density wounding method. This method was used to enhanced the numbers of keratinocyte cells in proximity to the scratch wound. (A) Pipette tips were attached to a multichannel pipettor and make parallel scratch wounds upon cells in a six well culture dish. Maintaining constant pressure, the tips were drawn across the well once in both horizontal and then in vertical directions. (B) The HDW method created a cross pattern of regularly spaced wounds in the cultured cells. The high density wounding (HDW) served to enrich the number of leader cells that function in the initiation of KCCM. The scale bar is 200 micron.

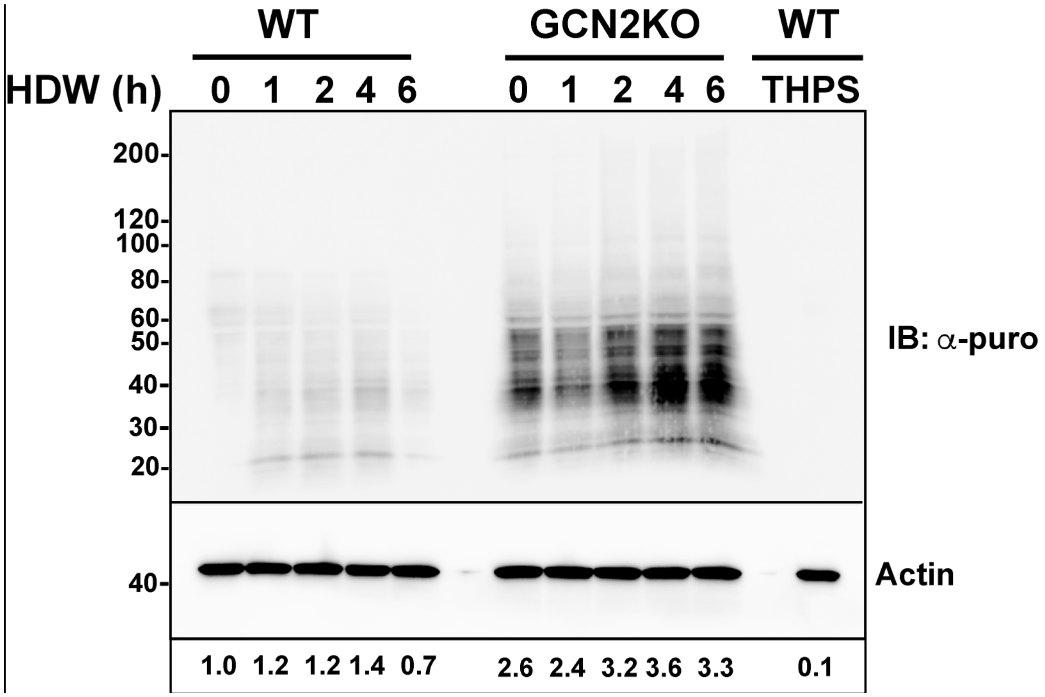

Supplemental Figure 2. Measurement of protein synthesis in WT and GCN2KO in response to HDW. WT and GCN2KO NTERT cells were collected following HDW for up to 6 h or no wounding (0). Thirty minutes prior to harvest, 1 μM puromycin was added to the cultures to label nascent polypeptides. Lysates were then prepared and separated by SDS-PAGE. Puromycin-labeled nascent polypeptides were visualized by immunoblot analysis using antibodies specific to puromycin. As a control, WT cells were treated with 1 μM thapsigargin (THPS) for 6 h in the absence of HDW. Levels of puromycin tagged proteins in the immunoblots are shown relative to WT cells not subjected to HDW. An immunoblot analysis of actin is shown in the bottom panel to illustrate that equal amounts of protein were analyzed for each lysate preparation. Molecular weight markers are indicated in KDal. This is a representative blot of three independent experiments with collective results shown in collectively shown in figure 2C.

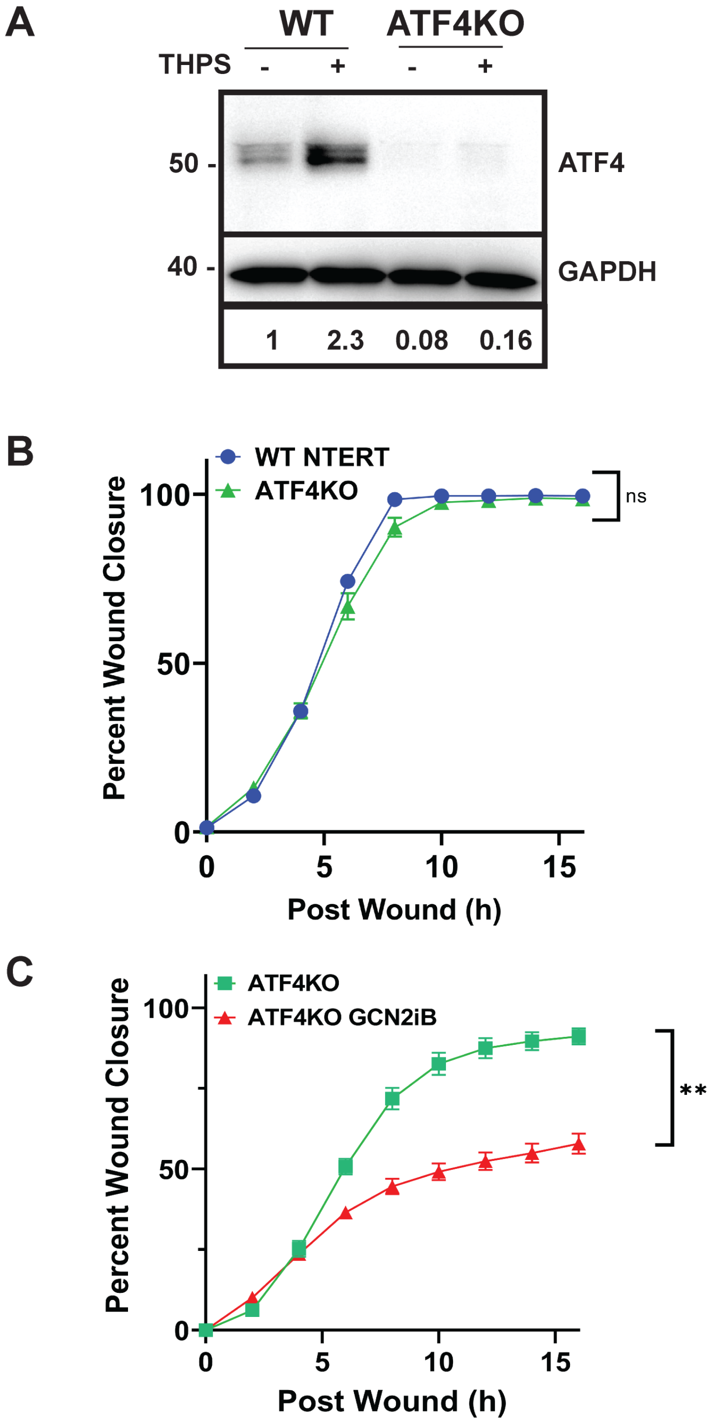

Supplemental Figure 3. Depletion of ATF4 in NTERT keratinocytes does not impair KCCM. (A) WT and ATF4KO cells were treated with 1 μM thapsigargin (THPS) for 6 h and the levels of ATF4 protein were measured by immunoblot analyses. (B) WT and ATF4KO cells were analyzed for closure during wound healing. n.s. $p>0.05$ two-tailed paired T-test (n=8) (C) ATF4KO cells were treated at the time of wounding with 5 μM GCN2iB or vehicle and wound closure was measured with Incucyte ZOOM Live-Cell Analysis System. Two-tailed paired T-test (n=11), ** $p<0.01$, error bars are SD.

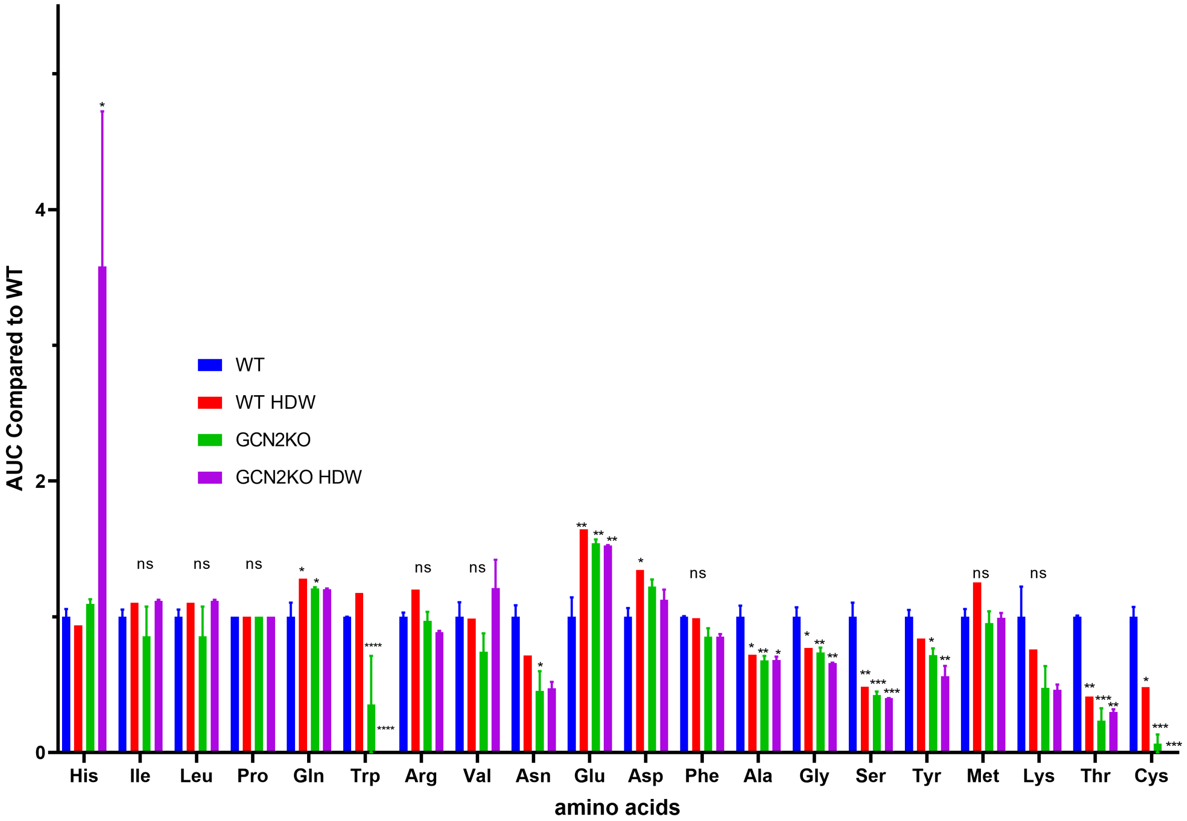

Supplemental Figure 4. Measurements of free amino acids in unwounded WT and GCN2KO cells and 4 h after wounding. The bar graphs show the area under the curve (AUC) values for the indicated amino acids in unwounded and wounded WT and GCN2KO cells relative to WT cells not subject to wounding. One-way ANOVA Dunnet's multiple comparisons were used for statistical analysis and each measurement was derived from 3 biological replicates. The error bars represent the standard error (SEM) from the mean. * $p<0.05$, ** $p<0.01$, *** $p<0.001$, and **** represents $p<0.0001$. ns indicates no significant difference.
